# Antibody dependent cell-mediated cytotoxicity selection pressure induces diverse mechanisms of resistance

**DOI:** 10.1080/15384047.2023.2269637

**Published:** 2023-10-25

**Authors:** David J. Zahavi, Rossin Erbe, Yong-Wei Zhang, Theresa Guo, Zoe X. Malchiodi, Rachael Maynard, Alexander Lekan, Rosa Gallagher, Julia Wulfkuhle, Emanuel Petricoin, Sandra A. Jablonski, Elana J. Fertig, Louis M. Weiner

**Affiliations:** a Department of Oncology and Lombardi Comprehensive Cancer Center, Georgetown University Medical Center, Washington, USA; b Department of Oncology, Johns Hopkins University School of Medicine, Baltimore, USA; c Department of Oncology, UC San Diego School of Medicine, San Diego, USA; d Center for Applied Proteomics and Molecular Medicine, George Mason University, Fairfax, USA

**Keywords:** Antibody-dependent cell-mediated cytotoxicity, natural killer cells, immunotherapy resistance, antibody target, cell surface, STAT1, immunoproteasome

## Abstract

Targeted monoclonal antibody therapy has emerged as a powerful therapeutic strategy for cancer. However, only a minority of patients have durable responses and the development of resistance remains a major clinical obstacle. Antibody-dependent cell-mediated cytotoxicity (ADCC) represents a crucial therapeutic mechanism of action; however, few studies have explored ADCC resistance. Using multiple *in vitro* models of ADCC selection pressure, we have uncovered both shared and distinct resistance mechanisms. Persistent ADCC selection pressure yielded ADCC-resistant cells that are characterized by a loss of NK cell conjugation and this shared resistance phenotype is associated with cell-line dependent modulation of cell surface proteins that contribute to immune synapse formation and NK cell function. We employed single-cell RNA sequencing and proteomic screens to interrogate molecular mechanisms of resistance. We demonstrate that ADCC resistance involves upregulation of interferon/STAT1 and DNA damage response signaling as well as activation of the immunoproteasome. Here, we identify pathways that modulate ADCC sensitivity and report strategies to enhance ADCC-mediated elimination of cancer cells. ADCC resistance could not be reversed with combinatorial treatment approaches. Hence, our findings indicate that tumor cells utilize multiple strategies to inhibit NK cell mediated-ADCC. Future research and development of NK cell-based immunotherapies must incorporate plans to address or potentially prevent the induction of resistance.

## Introduction

Tumor antigen-targeted monoclonal antibody (mAb) therapy has been established as a successful immunotherapy treatment modality alongside chemotherapy and radiotherapy for both hematological malignancies and solid tumors. The mAbs cetuximab and trastuzumab, which target epidermal growth factor receptor (EGFR) and human epidermal growth factor receptor 2 (HER2), respectively, are clinically effective in the treatment of a variety of cancers and see widespread use.^1–4^ However, only a minority of patients have durable responses, with the vast majority developing refractory disease within one year.^5,6^ While both cetuximab and trastuzumab are known to mediate their anti-tumor effects via several mechanisms, including inhibition of target receptor signaling, antibody dependent cell-mediated cytotoxicity (ADCC) has been demonstrated to be a pivotal *in vivo* mechanism.^7–12^ Natural killer (NK) cells, which express the FcγRIII low-affinity receptor for IgG – CD16, play a major role in tumor antigen-targeted mAb immunotherapy as mediators of ADCC.^13^ Despite the evidence suggesting ADCC plays a crucial role in the clinical responses to mAb therapy, prior research into therapeutic resistance has focused primarily on innate resistance or been restricted to mechanisms involving the signaling pathways associated with the growth factor receptor targets.^14–17^ Relatively few studies have incorporated effector cells to directly examine mechanisms underlying ADCC resistance.^18^ There is an urgent need for additional investigation of ADCC resistance using relevant models. Recently, rapid development of second-generation mAbs engineered with properties that enhance ADCC has led to improved results in clinical trials; however, resistance remains a major obstacle to clinical benefit.^19^

In order to further explore tumor cell-based mechanisms of resistance to ADCC our laboratory previously developed an *in vitro* model system for generating ADCC-resistant clones utilizing the EGFR-overexpressing squamous cell carcinoma cell-line A431, cetuximab, and the KIR-deficient and CD16V transduced NK cell line NK92-CD16V.^20^ Utilizing an *in vitro* model employing this NK92 cell line allowed for scalability, ease of manipulation, interrogation of specific resistance mechanisms that are not complicated by donor NK cell variations, and the study of multiple timepoints during the acquisition of resistance. We identified a novel resistance mechanism involving the loss of expression of multiple cell surface molecules that contribute to NK cell-target cell immune synapse formation in a single tumor cell-line model.^21^ Past studies of ADCC resistance have been limited by the use of models restricted to a single cancer type; this has made it difficult to translate pre-clinical data into meaningful therapeutic interventions. In fact, there remains no consensus on whether the level of expression of the mAb target is relevant to clinical efficacy or plays a role in resistance.^22–25^ We hypothesized that because many mAbs can act via ADCC, the underlying mechanisms of therapeutic resistance would be shared across different cancer types and targets. Thus, understanding common mechanisms of resistance applicable to multiple cancer contexts would be critical for developing strategies to overcome clinical resistance and improve the efficacy of mAb therapy.

In the present study, we generated ADCC-resistant variants of multiple cell lines representing diverse cell lineages utilizing our previously described *in vitro* model system. We demonstrate that irrespective of monoclonal antibody or target, ADCC resistance is characterized by alterations in the cell surface proteome that result in a loss of NK cell conjugation. We employed single-cell cellular indexing of transcriptomes and epitopes by sequencing (scCITE-seq) and single-cell assay for transposase-accessible chromatin using sequencing (scATAC-seq) to uncover molecular mechanisms that contribute to the development of ADCC resistance and identify potential biomarkers of resistance. We present evidence that although ADCC resistance is marked by a shared phenotype, cell-type intrinsic features result in diverse underlying mechanisms of resistance. Furthermore, our findings indicate that ADCC resistance is the result of activation of multiple molecular pathways and is unlikely to be reversed by targeting any single mechanism.

## Results

### ADCC selection pressure induces a shared resistance phenotype

**Figure 1.**
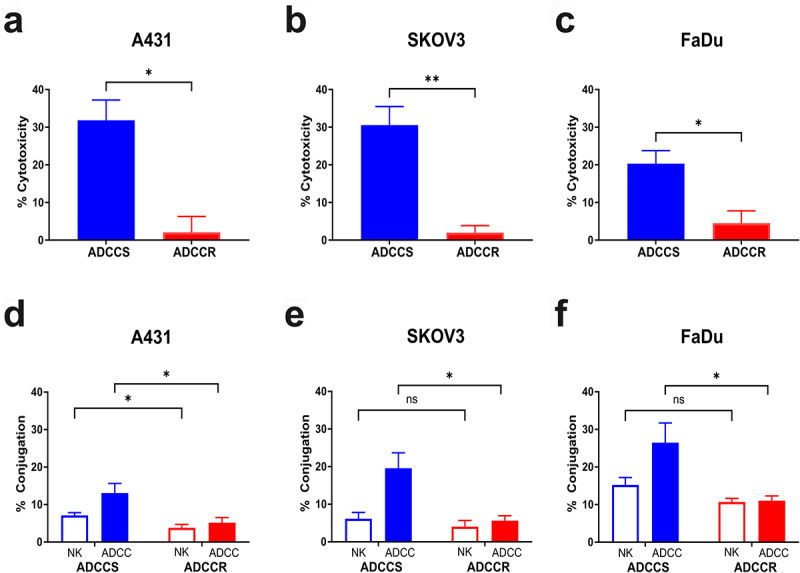
ADCC resistance in multiple cell line models is characterized by loss of NK cell conjugation (a-c), percent cytotoxicity of ADCC-sensitive (ADCCS, blue) compared to ADCC-resistant (ADCCR, red) A431 (a), SKOV3 (bB), and FaDu (c) cells as measured by ADCC assay (*n* = 3). Unpaired two-tailed *t-*test, *, *P*<.05, **, *P*<.01. Error bars, SEM. D-F, percent of NK92-CD16V cell-target cell conjugates in ADCC-sensitive (ADCCS, blue) compared to ADCC-resistant (ADCCR, red) A431 (d), SKOV3 (e), and FaDu (f) cells when treated with NK92-CD16V cells alone (open bar) or ADCC conditions (full bar) after 2hrs (*n* = 5 for A431, *n* = 4 for SKOV3 and FaDu). Unpaired two-tailed *t-*test, *, *P*<.05, ns, not significant. Error bars, SEM.

Previously, we used our model system to generate an ADCC-resistant variant of the EGFR-overexpressing squamous cell carcinoma cell-line A431 by continuously challenging cells with three day cycles consisting of the addition of fresh IL-2 stimulated NK92-CD16V cells at a 1:1 effector: target ratio and cetuximab at 1 μg/ml.^21^ Following 35 cycles of challenge, the A431 cells treated with ADCC conditions displayed complete resistance to ADCC, slower proliferation *in vitro* and *in vivo*, reduced EGFR expression, and loss of NK cell conjugation. In order to explore underlying mechanisms of resistance to ADCC that may be shared across cancer type and antibody target, we used our model system to re-derive ADCC-resistant A431 cells and generate additional ADCC-resistant variants using the HER2-overexpressing ovarian carcinoma cell-line SKOV3 and the EGFR-overexpressing head and neck squamous cell carcinoma cell line FaDu. Conditions were selected that produced >90% killing during a 72 hr challenge and similar levels of cytotoxicity in all cell lines as measured with our 4 hr ADCC assay and were cycles consisting of the addition of fresh IL-2 stimulated NK92-CD16V cells at a 3:1 effector: target ratio and trastuzumab at 5ug/ml for SKOV3 cells or cetuximab at 1ug/ml for FaDu cells, respectively. During the derivation of resistance, ADCC sensitivity was measured every five challenge cycles and both SKOV3 and FaDu cells acquired resistance in a similar time course as the original A431 cell line (Supplementary Fig. S1A and S1B). Challenge conditions using the NK92-CD16V cells alone or antibody alone had no effects on ADCC sensitivity over time (Supplementary Fig. S1A and S1B). At challenge cycle 35 there was a significant difference in ADCC sensitivity between the contemporaneously cultured untreated parental cells (ADCCS) and ADCC condition challenged cells (ADCCR) of each cell line signifying the acquisition of ADCC resistance (Figure 1a-c). As with the original A431 model, each ADCC-resistant cell line was characterized by a significant loss of NK cell conjugation compared to their sensitive counterparts (Figure 1d-e). Further initial phenotypic characterization revealed variable changes in morphology and proliferation *in vitro* that were cell line dependent. ADCC-resistant A431 cells did not display altered morphology but had a significantly reduced proliferative rate (Supplementary Fig. S1C and S1D). Interestingly, ADCC-resistant SKOV3 cells did not exhibit any changes in morphology or proliferation (Supplementary Fig. S1E and S1F) whereas ADCC-resistant FaDu cells displayed a modified morphology characterized by dense clumping of cells but no change in their proliferative rate (Supplementary Fig. S1G and S1H).

## Variable influence of antibody target expression or NK cell source on ADCC resistance

**Figure 2.**
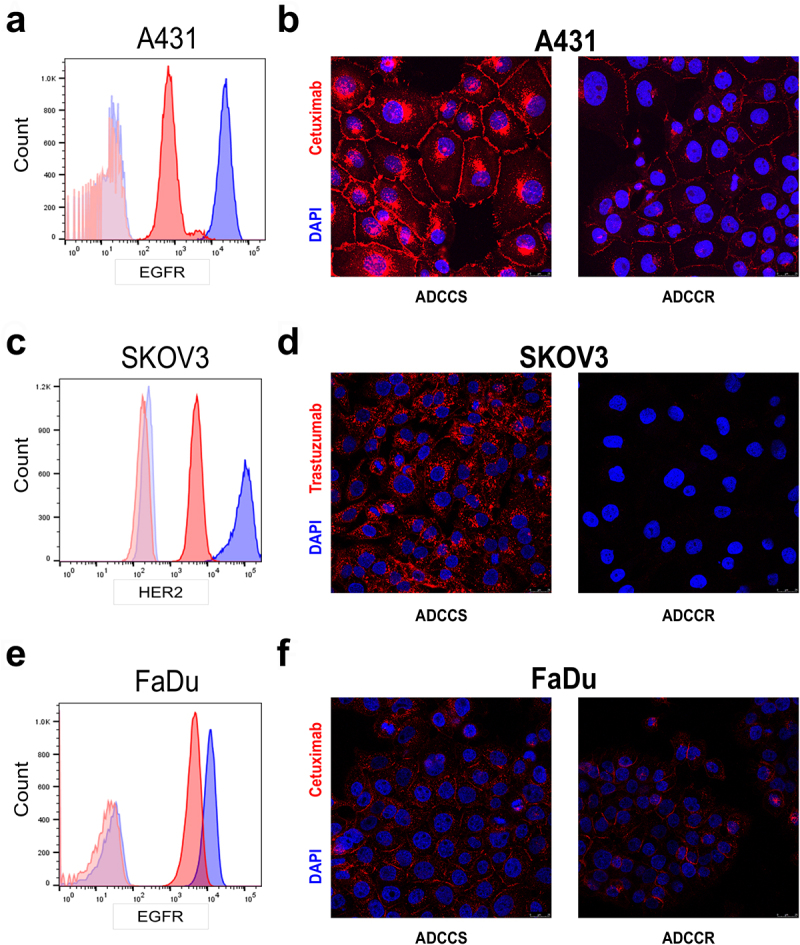
Variable loss of antibody target in ADCC resistance (a), surface EGFR expression measured by flow cytometry in ADCC-sensitive (blue) and ADCC-resistant (red) A431 cells with isotype control staining in ADCC-sensitive (light blue) and ADCC-resistant (light red) cells. Representative histogram from *n* = 3 experiments. (b), Representative immunofluorescence images of ADCC-sensitive (ADCCS) compared to ADCC-resistant (ADCCR) A431 cells incubated with Dylight550-conjugated cetuximab for 4hrs; red: cetuximab and blue: DAPI. Representative of *n* = 2, magnification 40×, scale bar = 25 μm. (c), surface HER2 expression measured by flow cytometry in ADCC-sensitive (blue) and ADCC-resistant (red) SKOV3 cells with isotype control staining in ADCC-sensitive (light blue) and ADCC-resistant (light red) cells. Representative histogram from *n* = 3 experiments. (d), Representative immunofluorescence images of ADCC-sensitive (ADCCS) compared to ADCC-resistant (ADCCR) SKOV3 cells incubated with Dylight550-conjugated trastuzumab for 3hrs; red: trastuzumab and blue: DAPI. Representative of *n* = 2, magnification 40×, scale bar = 25 μm. (e), surface EGFR expression measured by flow cytometry in ADCC-sensitive (blue) and ADCC-resistant (red) FaDu cells with isotype control staining in ADCC-sensitive (light blue) and ADCC-resistant (light red) cells. Representative histogram from *n* = 3 experiments. (f), Representative immunofluorescence images of ADCC-sensitive (ADCCS) compared to ADCC-resistant (ADCCR) FaDu cells incubated with Dylight550-conjugated cetuximab for 3hrs; red: cetuximab and blue: DAPI. Representative of *n* = 2, magnification 40×, scale bar = 25 μm.

The relationship between the level of expression of the mAb target and ADCC sensitivity is complex and poorly understood. There are conflicting reports on the level of influence of the amount of EGFR and HER2 expression on ADCC sensitivity mediated by cetuximab and trastuzumab *in vitro*, and most clinical studies have demonstrated that mAb responses do not correlate to mAb target expression levels in tumors.^26^ Our previous work in the original A431 ADCC-resistant variant demonstrated that EGFR is significantly reduced on the cell surface, the protein level and gene expression level in resistant cells.^21^ Although the reduction in surface expression of EGFR contributed to ADCC resistance, it was not the sole determinant. We next sought to assess whether the loss of mAb target represented a major shared driver of ADCC resistance in our models. In the current study, we redemonstrated a reduction in EGFR surface expression in resistant A431 cells (Figure 2a). Immunofluorescence staining using fluorescently labeled cetuximab confirmed that while there was a relative decrease in EGFR surface expression compared to untreated sensitive cells, sufficient EGFR surface expression remained for detectable cetuximab binding, further demonstrating that the reduction in EGFR cannot fully explain the complete loss in ADCC sensitivity (Figure 2b). However, in ADCC-resistant SKOV3 cells, there was both a significant reduction in HER2 expression (Figure 2c) and a nearly complete loss in trastuzumab binding (Figure 2d). Interestingly, in the FaDu model there was a more modest reduction in EGFR surface expression (Figure 2e) and cetuximab binding remained mostly intact (Figure 2f). To confirm whether ADCC resistance was defined solely by the antibody target, we also measured ADCC sensitivity in each cell-line pair when employing an alternative ADCC target antigen on the tumor cells. We observed no differences in the surface expression of the alternative growth factor receptor in any ADCC-resistant cell line compared to its sensitive counterpart (Supplementary Fig. S2A-S2C). However, each ADCC-resistant variant was less sensitive to killing even when the alternative target and therapeutic antibody were employed (Supplementary Fig. S2D). Thus, despite a shared phenotype of loss of NK cell conjugation, the level of expression of the mAb target has variable influence on ADCC sensitivity in a cell line-dependent manner, and loss of this expression is not the sole driver of resistance.

Our model system utilizes the KIR-deficient and high-affinity CD16 NK92 cell line to generate the ADCC-resistant variants. In order to confirm that the resistance phenotype was also applicable to varying NK effector cell populations, we measured the ADCC sensitivity of each cell line to cultured NK cells from five healthy donors. As expected, we observed high variability in the cytotoxic capabilities of NK cells from each donor. Crucially, we observed an overall trend of reduced ADCC sensitivity in each resistant variant to donor NK cells and a significant loss of ADCC activity of donor NK cells against the SKOV3 resistant cell line (Supplementary Fig. S2E). These findings suggest that, while NK cell expression of KIRs and other conjugation factors influence ADCC sensitivity, the resistance phenotype is not specific to NK92 cells but rather is applicable to normal NK cell populations.

## ADCC resistant cells do not activate alternative survival pathways

We next evaluated how each ADCC-resistant cell line adapted to changes in growth factor receptor expression and whether activation of alternative signaling pathways represented a possible shared molecular mechanism or biomarker of resistance. In ADCC-resistant A431 and SKOV3 cells there is a significant loss of EGFR and HER2 gene expression, respectively (Supplementary Fig. S3A). Acquired resistance to cetuximab and trastuzumab often involves alterations in intracellular signaling pathways in tumor cells that confer maintenance of proliferation despite the loss of their growth factor receptors and decreased susceptibility to immune effector-mediated cytotoxicity.^27,28^ Cell plasticity and epithelial-to-mesenchymal transition (EMT) is established as an important immune evasion mechanism that can be induced by treatment with mAbs and has also been shown to regulate sensitivity to NK cell killing via ICAM1.^29–31^ Interrogation of EMT markers revealed no induction of transition between cell states in any of the ADCC-resistant cell lines relative to their parental untreated cell line (Supplementary Fig. S3B). We next investigated the major effector pathways downstream of EGFR and HER2 signaling in each ADCC-sensitive and -resistant cell-line pair. A431 ADCC-resistant cells had marked reductions in total EGFR protein expression and EGFR phosphorylation as well as slight reductions in ERK and AKT signaling in concordance with their reduced proliferative rate (Supplementary Fig. S3C). While SKOV3 ADCC-resistant cells had a similar loss of total HER2 and phospho-HER2 expression, there was maintenance of downstream ERK and AKT signaling levels (Supplementary Fig. S3D). ADCC-resistant FaDu cells displayed a distinct signature of increased ERK signaling and decreased AKT signaling (Supplementary Fig. S3E). To further explore alternative signaling adaptations, RPPA was performed on each cell-line pair for canonical growth factor signaling pathways (Suplementary Table 1). There was no induction of alternative signaling pathways, including HER3, IGF-1 R, ALK, mTOR, PTEN, MEK, or MET in any of the resistant cell lines (Supplementary Fig. S3F-S3H). The protein and phospho-protein levels found by the RPPA-based signaling analysis were concordant with those from western blotting and revealed diverse signaling adaptations to ADCC selection pressure based on cell lineage. Taken together, these results suggest that tumor cells employ a multitude of mechanisms to overcome growth factor signaling inhibition in the context of immune attack.

## ADCC resistant cells are characterized by altered cell surface proteomes

**Figure 3.**
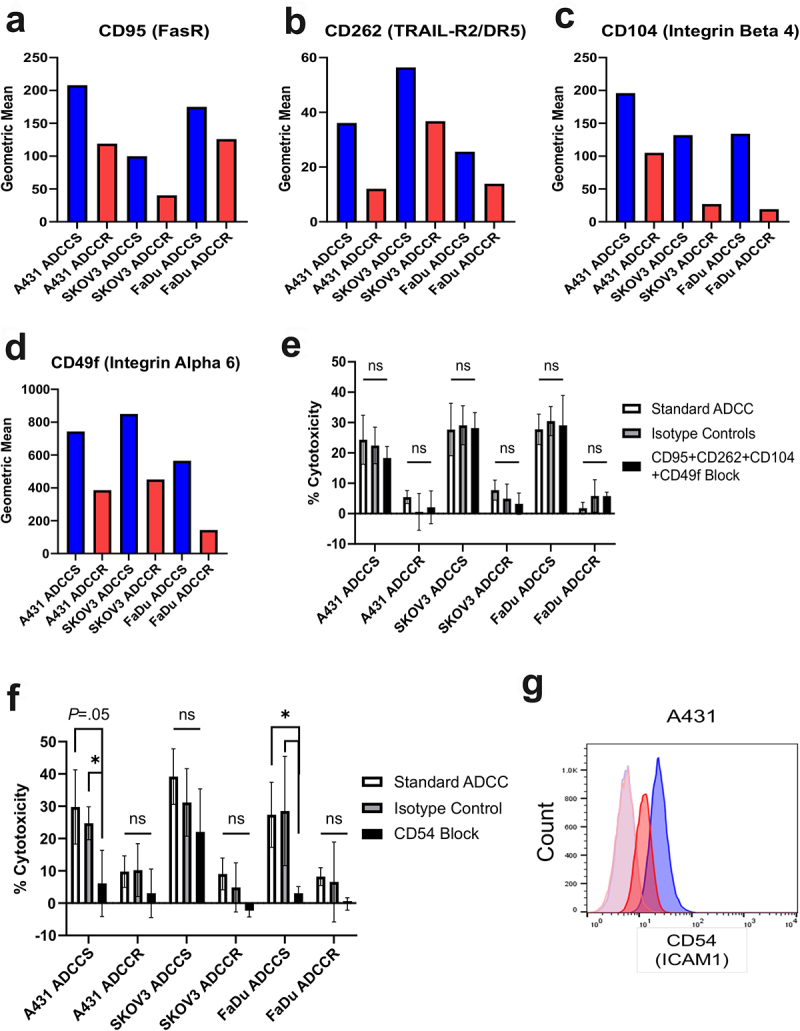
ADCC resistance is associated with loss of cell surface proteins involved in immune synapse formation (a-d), geometric mean of CD95 (FasR) (a), CD262 (TRAIL-R2/DR5) (b), CD104 (Integrin beta 4) (c), and CD49f (Integrin Alpha 6) (d) expression as measured by BioLegend LEGENDScreen™ in ADCC-sensitive (blue) compared to ADCC-resistant (red) cells. (e), percent cytotoxicity of each ADCC-sensitive and ADCC-resistant cell line as measured by ADCC assay when untreated (white bar), pretreated with a combination of 10 µg/ml of each isotype control antibody (gray bar), or pretreated with a combination of 10 µg/ml of each blocking antibody for CD95, CD262, CD104, and CD49f (black bar) for 1 hr (*n* = 4). Unpaired two-tailed *t*-test, ns, not significant. Error bars, SEM. F, percent cytotoxicity of each ADCC-sensitive and ADCC-resistant cell line as measured by ADCC assay when untreated (white bar), pretreated with 10 µg/ml of isotype control antibody (gray bar), or pretreated with 10 µg/ml of blocking antibody for CD54 (black bar) for 1 hr (*n* = 3). Unpaired two-tailed *t*-test, *, *P*<.05, ns, not significant. Error bars, SEM. G, Representative flow cytometry analysis of CD54 (ICAM1) in ADCC-sensitive (blue) and ADCC-resistant (red) A431 cells with isotype control staining in ADCC-sensitive (light blue) and ADCC-resistant (light red) cells.

Our initial studies of ADCC-resistant A431 cells uncoveredthat a cell surface signature comprised the downregulation of many proteins involved in cell adhesion is associated with resistance.^21^ Given the shared ADCC resistance phenotype of loss of NK cell conjugation, we next sought to identify if a similar cell surface signature was shared by all three ADCC resistance models. We employed the LEGENDScreen™ antibody panel for 361 cell surface markers to establish comprehensive cell surface mapping of each cell line (Supplementary Table 2). Notably, there was shared loss of four cell surface proteins by all ADCC-resistant cell lines: CD95 (Fas receptor), CD262 (TNF-related apoptosis-inducing ligand-receptor 2 [TRAIL-R2] aka death receptor 5 [DR5]), CD104 (Integrin Beta 4), and CD49f (Integrin Alpha 6) (Figure 3a-d). The engagement of Fas ligand and TRAIL ligands expressed by NK cells and their respective death receptors FasR and TRAIL-R2 are known mechanisms of NK cell-mediated ADCC.^32^ There have also been reports that CD104 and CD49f, which complex together to form the laminin receptor, play a role in NK cell recognition, conjugation, and lysis.^33,34^ The loss of these cell surface proteins in all tested ADCC- resistant cell lines suggested a potential shared mechanism underlying the loss of NK cell conjugation. Consequently, we hypothesized that the blockade of these interactions in sensitive cells could recapitulate resistance. We measured ADCC cytotoxicity of each cell line in the presence or absence of concomitant blockade of CD95, CD262, CD104, and CD49f and found that this was unable to modulate sensitivity to ADCC (Figure 3e). Therefore, the loss of these markers is associated with resistance but does not drive the phenotype.

The initiation of NK-cell effector functions, including ADCC, requires successful formation of an immunological synapse between the NK cell and its target cell.^35^ Many surface proteins involved in cell adhesion also play a role in the formation of the immune synapse for NK cell-mediated ADCC such as CD54 (ICAM1), CD81, CD58, CD56, and CD44; however the binding of LFA-1 expressed by NK cells to CD54 expressed by target cells is considered the most crucial.^36,37^ We measured ADCC sensitivity of each cell line in the presence or absence of blockade of CD54 and confirmed in our A431 and FaDu models that CD54 interactions are essential for ADCC (Figure 3f); and trastuzumab-mediated ADCC of SKOV3 cells was minimally affected by CD54 blockade in agreement with previous studies.^38^ Importantly, CD54 surface expression is reduced in A431 ADCC-resistant cells and represented a potential cell lineage-specific resistance mechanism (Figure 3g). Therefore, we hypothesized that re-expressing CD54 in the A431 ADCC-resistant line could restore NK cell conjugation and ADCC sensitivity. We found that transduction of A431 ADCC-resistant cells with CD54 expression plasmid resulted in restoration of CD54 protein and cell surface expression to similar levels as the sensitive parental cells (Supplementary Fig. S4B and S4C). Despite the re-expression of CD54, ADCC-resistant A431 cells retained their phenotype of reduced NK92-CD16V cell conjugation and insensitivity to cytotoxicity (Supplementary Fig. S4D and S4E).

## Single-cell RNA and protein sequencing with scCITE-seq identifies ADCC resistance gene signature

**Figure 4.**
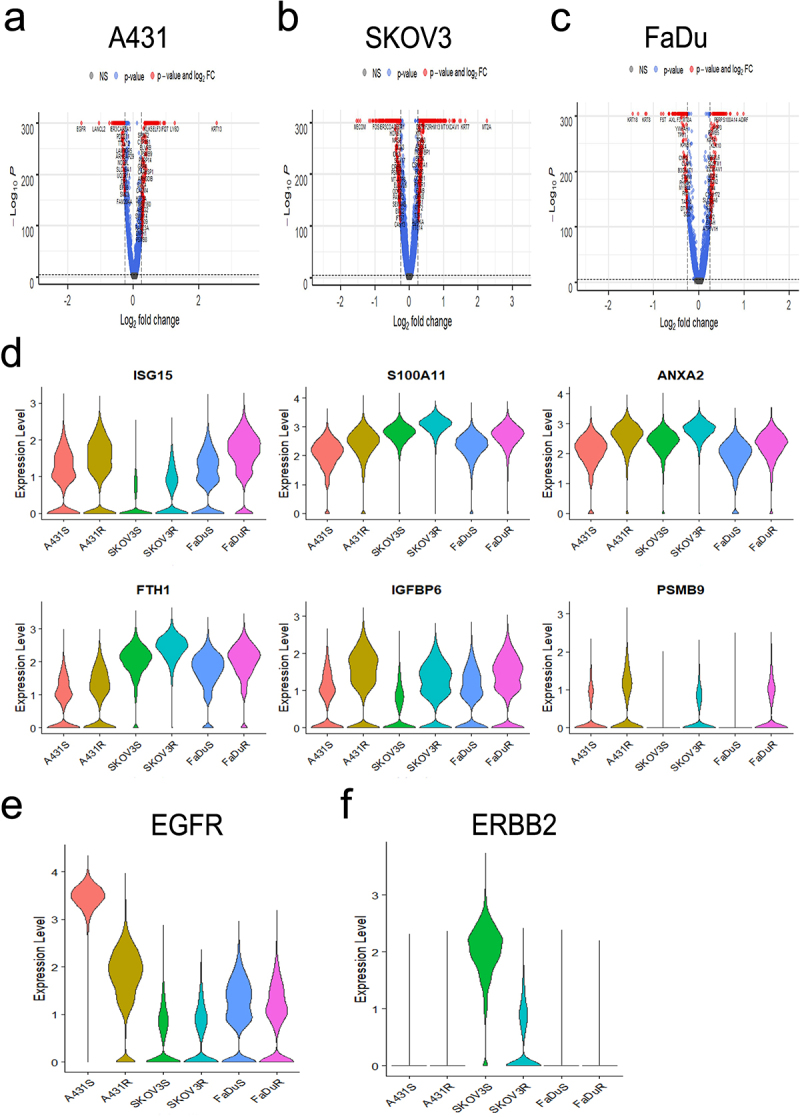
Single-cell CITE-seq identifies ADCC resistance associated genes A-C, volcano plots of genes differentially expressed between A431 (a), SKOV3 (b), and FaDu (c) ADCC-sensitive and ADCC-resistant cells. P-value was calculated by Wilcoxon rank-sum test and then adjusted by Bonferroni correction. Genes with – Log10(p-value)>1.3 are marked in blue and genes with both – Log10(p-value)>1.3 and log2 fold change (FC) ±0.5 were considered significant and are marked in red. NS, not significant. D, violin plots of the gene expression level in each cell line for the six genes with shared statistically significant upregulation in all ADCC-resistant cell lines. E and F, violin plot of *EGFR* (E) and *ERBB2* (F) gene expression level in each ADCC-sensitive and -resistant cell line.

In order to evaluate molecular mechanisms of ADCC resistance and interrogate heterogeneity in resistant cell populations, we performed scCITE-seq on each ADCC-sensitive and -resistant cell-line pair. Each cell line received one additional challenge cycle of either medium alone (untreated; sensitive cell lines) or ADCC conditions (ADCC challenged; resistant cell lines), were subsequently verified for phenotype, and after a total of 9 days were collected in single-cell suspensions for the library preparations and sequencing. UMAP clustering of the RNA expression data showed each cell line and their respective sensitive and resistant variants cluster independently (Supplementary Fig. S5A). Differential gene expression analysis of each cell-line pair revealed that 323 genes in A431, 707 genes in SKOV3, and 293 genes in FaDu ADCC-resistant cells were differentially expressed (Figure 4a-c). Our previously published gene expression analysis of ADCC-sensitive and -resistant A431 cells utilizing the Coordinated Gene Activity in Pattern Sets (CoGAPS) algorithm and PatternMarker statistic for CoGAPS identified a pattern of 300 genes up-regulated in resistant cells (Pattern 3) that was composed of clusters of interferon-associated and histone-associated genes.^21^ In the current study, we observed that this gene signature was also associated with each ADCC-resistant cell-line variant (Supplementary Fig. S5B). We next sought to identify specific genes with upregulation in every ADCC-resistant cell line to uncover possible shared molecular mechanisms and pathways underlying resistance. Six genes were upregulated in all tested ADCC-resistant cell lines: *ISG15*, *S100A11*, *ANXA2*, *FTH1*, *IGFBP6*, and *PSMB9* (Figure 4d). We validated the increase in *PSMB9* gene expression in each ADCC resistant variant using RT-qPCR (Supplementary Fig. S5C). STRING protein–protein interaction network analysis was used to identify whether the genes with significantly upregulated expression represented a common significantly affected pathway. We found 4 of the 6 gene products converged on the immunoproteasome, of which PSMB9 is a component (Supplementary Fig. S5D).

To further confirm the contributions of the mAb target to resistance and possible heterogeneity within each resistant cell population our scCITE-seq profiling included antibodies for EGFR and HER2 to concurrently and quantitatively measure both the transcript and surface protein expression levels of these genes. We confirmed that at the RNA level, there is a global loss of EGFR gene expression in A431 but not FaDu ADCC-resistant cells and a loss of HER2 gene expression in SKOV3 ADCC-resistant cells (Figure 4e,f). Examination of surface protein expression revealed more similar levels of heterogeneous surface expression of EGFR and HER2 between each ADCC-sensitive and -resistant cell-line pair; this again suggests that ADCC resistance and the lack of NK cell conjugation cannot be fully explained by a loss of mAb target expression (Supplementary Fig. S5E and S5F).

## Single-cell ATAC sequencing reveals epigenetic mechanisms underlying ADCC resistance

Our previous interrogation of ADCC resistance in the A431 cell line uncovered differential expression of many histone-associated genes^21^, which implicated epigenetic modulation as a possible mechanism of resistance. We used scATAC-seq to further examine the epigenetic alterations present in ADCC-resistant cells and determine whether genes with differential gene expression were regulated at the chromatin level. Chromatin accessibility scores for each gene were calculated for each cell-line pair and revealed that 1799 genes in A431, 5236 genes in SKOV3, and 2351 genes in FaDu ADCC-resistant cells respectively, had significant alterations in accessibility (Supplementary Fig. S6A-S6C). We next sought to identify genes that had both differential expression and accessibility in each cell-line pair and found a 25.4% overlap for A431, 49.9% for SKOV3, and 35.2% for FaDu, respectively. This large proportion of coordinate changes between gene expression and chromatin accessibility is consistent with our previous hypothesis of epigenetic regulation of this resistance mechanism suggested by the RNA data alone in our previous study. We subsequently examined whether any of the genes with shared differential expression across all ADCC-resistant cell lines also had shared differential accessibility. Six hundred and forty-four genes had a shared increase in accessibility and 868 genes had a shared decrease (Supplementary Fig. S6D). Of the six genes with upregulated gene expression in all ADCC-resistant cell lines only *S100A11* was associated with a significant increase in chromatin accessibility in each cell-line pair, however this finding was lost when comparing all ADCC-resistant and -sensitive cell lines in aggregate. Furthermore, we used this method to investigate whether the antibody target expression was regulated at the epigenetic level. Notably, in the A431 ADCC-resistant cell line there was a significant decrease in chromatin accessibility score for *EGFR* compared to sensitive cells.

## Interferon response pathways are associated with ADCC resistance

**Figure 5.**
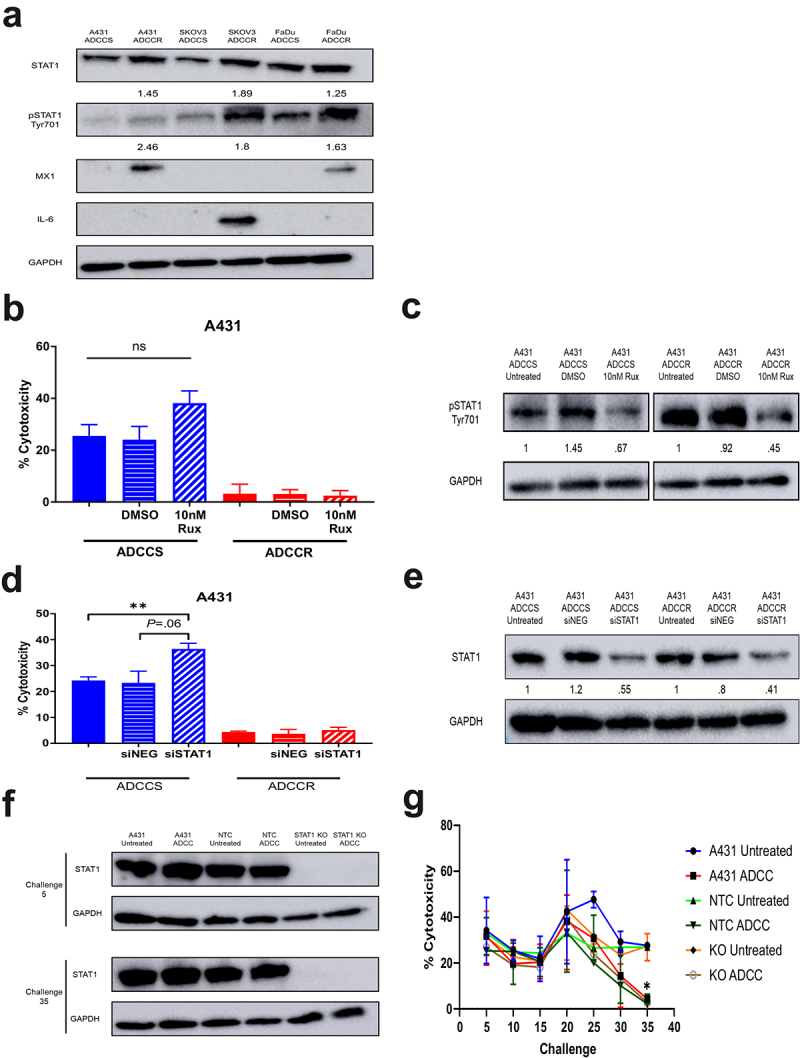
STAT1 signaling is associated with but does not drive acquisition of ADCC resistance (a), western blot analysis of STAT1 pathway proteins in ADCC-sensitive (ADCCS) and ADCC-resistant (ADCCR) A431, SKOV3, and FaDu cells. Densitometry values for expression normalized to GAPDH in ADCC-resistant relative to ADCC- sensitive cells are indicated. (b), percent cytotoxicity of ADCC-sensitive (blue) and ADCC-resistant (red) A431 cells as measured by ADCC assay when cells are incubated in either medium alone (solid bars) or medium supplemented with DMSO (horizontal hash bars) or 10 nM ruxolitinib (diagonal hash bars) for 72hrs prior to ADCC assay (*n* = 3). Unpaired two-tailed *t*-test, ns, not significant. Error bars, SEM. (c), Representative western blot analysis of phospho-STAT1 protein expression in ADCC-sensitive and ADCC-resistant A431 cells when cells are incubated in either medium alone (untreated) or medium supplemented with DMSO or 10 nM ruxolitinib for 72hrs. Densitometry values for expression normalized to GAPDH in cells treated with DMSO or ruxolitinib compared to untreated cells are indicated. (d), percent cytotoxicity of ADCC-sensitive (blue) and ADCC-resistant (red) A431 cells as measured by ADCC assay when cells are incubated in either medium alone (solid bars) or transfected with control scramble siRNA (siNEG, horizontal hash bars) or siRNA specific for STAT1 (diagonal hash bars) for 48 h prior to ADCC assay (*n* = 3). Unpaired two-tailed *t*-test, **, *P*<.01. Error bars, SEM. (e), Representative western blot analysis of STAT1 protein expression in ADCC-sensitive and ADCC-resistant A431 cells when cells are incubated in either medium alone (untreated) or transfected with control scramble siRNA (siNEG) or siRNA specific for STAT1 for 48hrs. Densitometry values for expression normalized to GAPDH in cells transfected with control scramble (siNEG) or siRNA specific for STAT1 compared to untreated cells are indicated. (f), western blot analysis of STAT1 protein expression in untreated and ADCC condition treated parental A431 cells, A431 cells transduced with a non-targeting control CRISPR-cas9 (NTC), and A431 cells transduced with CRISPR-cas9 specific for STAT1 (STAT1-KO) at the indicated challenges. (g), percent cytotoxicity of untreated and ADCC condition treated parental A431 cells, A431 cells transduced with a non-targeting control CRISPR-cas9 (NTC), and A431 cells transduced with CRISPR-cas9 specific for STAT1 (STAT1-KO) at every 5 challenges during derivation of resistance as measured by ADCC assay (*n* = 2). Unpaired two-tailed *t-*test, *, *P*<.05. Error bars, SEM.

Interferon (IFN)-induced STAT1 signaling is associated with tumor cell resistance to genotoxic stress and has been reported to regulate growth factor receptor expression.^39,40^ Additionally, overexpression of the IFN/STAT1 pathway has been shown to contribute to cetuximab therapy failure.^41^ Our previous analysis of A431 ADCC-resistant cells discovered activation of IFN response genes and overexpression of downstream signaling proteins including JAK1 and STAT1.^21^ Given that we found a shared upregulation of IFN-related genes in all ADCC-resistant cell lines, we focused our initial investigations on the STAT1 pathway. We observed increases in STAT1 expression and phospho-STAT1 activation/expression across all ADCC-resistant cell lines, indicating activation of STAT1 signaling (Figure 5a). This shared upregulation of IFN-induced STAT1 signaling led to divergent expression of downstream effector proteins such as IL-6 and M×1 (Figure 5A), both of which are known to mediate tumor cell resistance to immune effector cell-mediated attack.^42,43^ We examined whether inhibition of STAT1 signaling could enhance ADCC sensitivity and possibly reverse ADCC resistance. Pharmacological inhibition of JAK1/2 phosphorylation of STAT1 with ruxolitinib moderately augmented ADCC sensitivity in the sensitive parental A431 cells but was unable to restore activity in the resistant cells (Figure 5b,c). Knockdown of total STAT1 protein similarly amplified ADCC cytotoxicity only in the A431 ADCC-sensitive cell line (Figure 5d,e). We hypothesized that although STAT1 inhibition may not reverse the established resistance phenotype it could prevent the development of resistance. We performed ADCC challenges to derive resistance in contemporaneously cultured parental cells, non-targeting CRISPR-Cas9 control cells, and STAT1 CRISPR-Cas9 knockout A431 cells. The complete knockout of STAT1 was unable to modify the acquisition of resistance and all ADCC challenged lines once again became resistant by challenge 35 (Figure 5f,g). Hence, although IFN-induced signaling through STAT1 is a common feature of resistance, STAT1 signaling is not a driver of the resistance phenotype.

The DNA damage response (DDR) signaling pathway and associated phosphoinositide 3-kinase-like serine threonine kinases such as ataxia telangiectasia mutated (ATM) and ataxia telangiectasia and RAD3-related (ATR) are closely linked to NK cell-mediated killing and IFN responses.^44,45^ To refine the dynamic changes associated with resistance, we performed additional scRNA-seq of ADCC-sensitive parental A431 cells, A431 cells 24 h after a single cycle of ADCC challenge, and ADCC-resistant A431 cells to examine which genes maintain cell viability in response to ADCC conditions and the progression of gene expression changes. UMAP analysis identifies a shift in gene expression in the short-term challenge toward the resistant cellular population, suggestive of a global change in gene expression from ADCC selection pressure. A subset of the resistant cells cluster separately from the expression profiles of the challenged cells, suggestive of a phenotypic cell state transition associated with resistance in these cells (Supplementary Fig. S7A). Transfer learning from our resistance signature defined in bulk further confirms this observation, although we note a rare subpopulation of cells even in the sensitive group with this signature (Supplementary Fig. S7B). Pseudo-time analysis infers a trajectory associated with continuous cell state changes in the acquisition of resistance (Supplementary Fig. S7C), coordinated with the loss of EGFR expression in the cells (Supplementary Fig. S7D). Analyzing changes in DNA damage genes along the pseudotime trajectory indicated significant upregulation of DDR pathway genes such as *PARP1*, *STAT3*, *STAT1*, *TP53*, *ATM*, and *MDM2* during the acquisition of resistance in A431 cells (Supplementary Fig. S8A). We next confirmed that each ADCC-resistant cell line had increased ATM and ATR protein expression (Supplementary Fig. S8B). We hypothesized that targeting these DDR components might offer a rational way to manipulate ADCC activity. Due to the remarkable upregulation of ATR expression, we chose to focus the current study on the effects of ATR inhibition. Similar to STAT1 pathway inhibition, we observed that inhibition of ATR kinase activity could enhance cytotoxicity of ADCC-sensitive cells but could not reverse resistance in the A431 (Supplementary Fig. S8C and S8D) and SKOV3 (Supplementary Fig. S8E and S8F) cell lines. In contrast, ATR kinase inhibition was able to modestly restore sensitivity in ADCC-resistant FaDu cells but could not augment killing of sensitive cells (Supplementary Fig. S8G and S8H). While gene expression profiling and western blots showed increased expression of ATM and ATR, our RPPA analysis included measures of ATM and ATR phosphorylation/activation that suggest there may not be robust activation of the protein itself in all cell lines. This may be a likely explanation for the inability to restore ADCC sensitivity.

## ADCC selection pressure activates the immunoproteasome

**Figure 6.**
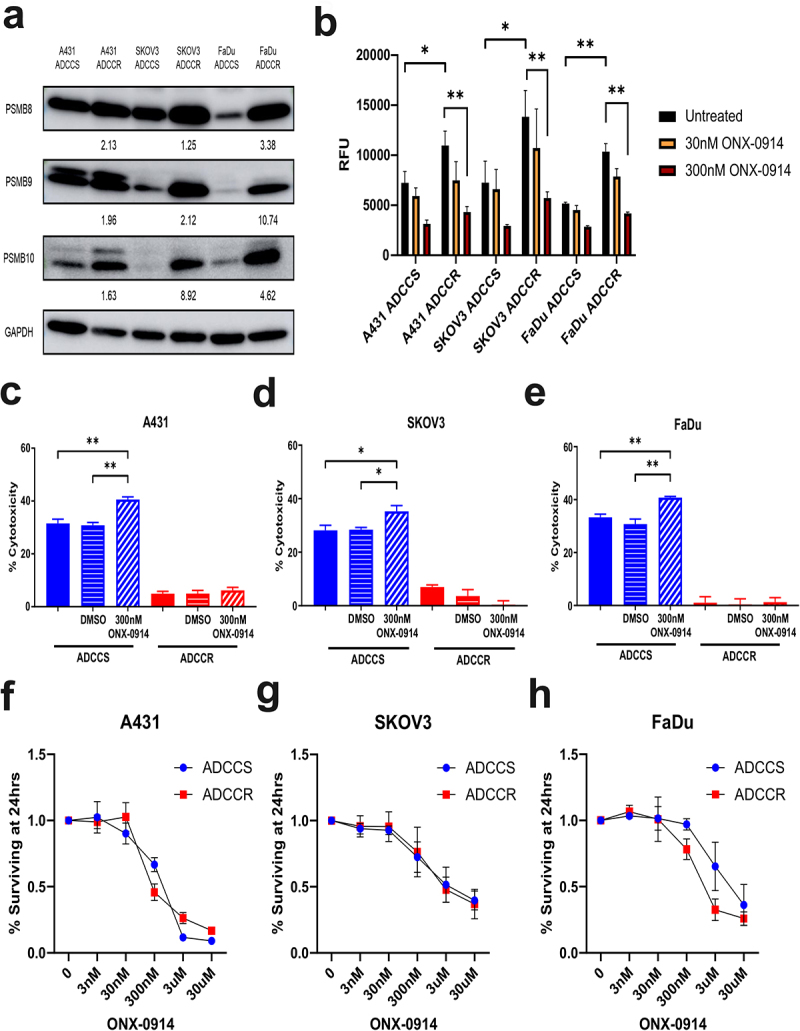
Immunoproteasome activation is associated with ADCC resistance (a), western blot analysis of immunoproteasome subunit expression in ADCC-sensitive (ADCCS) and ADCC-resistant (ADCCR) A431, SKOV3, and FaDu cells. Densitometry values for expression normalized to GAPDH in ADCC resistant relative to ADCC sensitive cells are indicated. (b), immunoproteasome activity in ADCC-sensitive (ADCCS) and ADCC-resistant (ADCCR) A431, SKOV3, and FaDu cells as measured by fluorometric plate based assay. Relative fluorescent units (RFU) were measured for cleavage of immunoproteasome specific substrate to 7-amino-4-methylcoumarin after 1 hr in the presence of absence of the immunoproteasome inhibitor ONX-0914. C-E, percent cytotoxicity of ADCC-sensitive (blue) and ADCC-resistant (red) A431 (c), SKOV3 (d), and FaDu (e) cells as measured by ADCC assay when cells are incubated in either medium alone (solid bars) or medium supplemented with DMSO (horizontal hash bars) or 300 nM of the immunoproteasome inhibitor ONX-0914 (diagonal hash bars) for 1 hr prior to ADCC assay (*n* = 3 for A431 and FaDu, *n* = 4 for SKOV3). Unpaired two-tailed *t*-test, *, *P*<.05, **, *P*<.01. Error bars, SEM. F-H, cell viability represented as the percent of cells remaining relative to the untreated ADCC-sensitive (blue) and ADCC-resistant (red) A431 (f), SKOV3 (g), and FaDu (h) cells as measured by crystal violet staining of cells after 24hrs of incubation in either medium alone (untreated) or medium supplemented with various concentrations of ONX-0914.

The immunoproteasome is an isoform of the constitutive 20S proteasome that becomes expressed when cells are stressed or in response to interferon-gamma and has recently become an attractive therapeutic target in some solid tumors.^46,47^ We observed protein expression increases in all three immunoproteasome subunits (PSMB8, PSMB9, and PSMB10) by each ADCC-resistant cell line (Figure 6a). These increases in subunit expression resulted in significant upregulation of immunoproteasome protein cleavage activity which could be inhibited by the irreversible immunoproteasome-specific compound ONX-0914 (Figure 6b). We first examined if ONX-0914 could modulate ADCC sensitivity and found that immunoproteasome inhibition could enhance killing of ADCC-sensitive but not resistant cells (Figure 6c,d). To further investigate whether ONX-0914 had potential as a combinatorial therapeutic strategy, we measured cell viability at a range of ONX-0914 concentrations to determine if ADCC-resistant cell lines were more susceptible to immunoproteasome inhibition. We found that ADCC-sensitive and -resistant cells had no significant differences in their response to ONX-0914 (Figure 6e-g).

## Discussion

This work builds on our previous exploration of tumor-cell-based resistance mechanisms to ADCC and represents the first comprehensive analysis of ADCC resistance mechanisms in multiple models. ADCC has been established as critical mechanism of action for targeted mAb cancer therapy and there is increasing interest in designing next-generation mAbs with enhanced ADCC activity.^19^ The majority of solid tumor patients treated with targeted mAbs have tumor progression within one year due to acquired resistance, and few studies have been designed to investigate ADCC specific mechanisms.^5,6,18^ We employed a consistent model of ADCC selection pressure using the NK92-CD16V cell line as effectors and a diverse array of target cell lines: A431, SKOV3, and FaDu. We found that ADCC selection pressure-induced resistance on a similar time course in each cell line and that, in comparison to ADCC-sensitive parental cells, each ADCC-resistant variant was characterized by reduced NK cell conjugation and upregulation of genes, proteins, and signaling pathways associated with interferon/STAT1, the DDR, and the immunoproteasome. We endeavored to uncover tumor cell-based molecular mechanisms to ADCC resistance that were shared across cancer type and mAb target to identify potentially high-impact therapeutic strategies. However, despite a shared ADCC resistance phenotype, each ADCC-resistant variant displayed distinct signaling adaptations, cell surface patterns, and IFN-response effectors. Taken together, our results suggest that cell lineage-intrinsic factors influence the responses to ADCC selection pressure and tumor cells possess a multitude of strategies to escape NK-cell mediated immune attack. Furthermore, the role of mAb target expression in ADCC sensitivity and resistance is also cell line-dependent. Our current work focused on tumor-cell-based mechanisms of resistance and future study will interrogate potential mechanisms involving the induction of changes in the effector cells such as exhaustion.

We demonstrated that STAT1, ATR, and immunoproteasome inhibition could all modulate ADCC sensitivity in naïve cell populations. However, none of the therapeutic interventions were able to reverse the resistance phenotype. We hypothesize that ADCC resistance is likely the end result of multiple mechanisms employed by tumor cells to survive immune attack and targeting any single mechanism will be unable to reestablish sensitivity. In addition, ADCC resistance is acquired over a long period of persistent selection pressure. In order to better interrogate drivers of the resistance phenotype, future studies will incorporate sequencing and profiling at multiple timepoints during the derivation of the resistant variants as well as a multi-omics approach. Since only a minority of patients have lasting responses to cetuximab and trastuzumab, it is critical to identify biomarkers of resistance, however to date few are known.^48,49^ IFN/STAT signaling is viewed as a controversial biomarker of response to immunotherapy^50^, although recent data from PD-L1 and PD-1 inhibitors in the neoadjuvant setting for breast cancer strongly implicated pre-treatment levels of phosphorylated STAT1 as predicting response to these agents.^51^ In addition, Samuels *et al*. showed that the expression of immunoproteasome subunits is a biomarker for response to checkpoint blockade.^52^ We demonstrated that ADCC resistance is marked by upregulation of IFN response genes and the immunoproteasome, but additional studies are needed to examine the potential role of IFN/STAT1 signaling and the immunoproteasome as biomarkers in the context of targeted mAb therapy in patients.

Finally, once established, the ADCC resistance phenotype is durable and transient assays may not be able to adequately evaluate the effects of therapeutic strategies and their possible relevance in the clinic. Furthermore, a limitation of our *in vitro* model system is that additional cell types and factors present in the tumor microenvironment that can play a role in ADCC resistance require future study. Upcoming work to establish mouse models of ADCC resistance will help address these issues. Importantly, our previous data in the A431 model showed that ADCC-resistant cells did revert to their original ADCC-sensitive phenotype when cultured in the absence of ADCC selection pressure for a length of time comparable to what is required to induce resistance.^21^ That finding, in addition to the resistance gene signature comprised histone-associated genes and scATAC-seq analysis identification of modulations to *EGFR* accessibility suggests a possible epigenetic link to resistance that needs to be further explored. While our results demonstrate ADCC resistance is not easily reversed, the best potential solution may be to prevent resistance from developing. Monoclonal antibodies are administered to patients on a continuous schedule, yet it remains unclear as to whether such a dosing regimen is necessary or more effective than an intermittent schedule.^53,54^ Our work highlights the need for additional investigation into targeted mAb dosing regimens; we hypothesize that an intermittent dosing regimen that allows for periods of rest in the absence of ADCC selection pressure could either delay or prevent the acquisition of ADCC resistance. Our future studies will explore the effect of modifying the challenge cycles to incorporate periods of rest on the acquisition of resistance.

In summary, the results highlighted in this paper demonstrate that ADCC resistance is multifactorial. Our model system employing the NK92-CD16V effector cell line allows for the specific examination of these evasion mechanisms, and the results extrapolate to human donor NK cells, supporting the biological relevance of our findings. It should be noted that NK92 cells are under investigation as a possible “off-the-shelf” immunotherapy for many solid tumors and also are in use as a chimeric antigen receptor (CAR) platform.^55,56^ We speculate that our findings will also have applicability to CAR-NK therapy, and that the continued study of ADCC resistance mechanisms will be of high relevance to the future advancement of NK92-based immunotherapy.

## Materials and methods

### Cell lines and culture

A431, SKOV3, and FaDu cell lines were obtained from the Georgetown Lombardi Tissue Culture Shared Resource (TCSR), and ADCC-sensitive and -resistant variants were routinely verified by DNA fingerprinting prior to downstream analysis. For all three cell lines, the culture conditions used were: High-glucose Dulbecco’s Modified Eagle Medium (DMEM) (HyClone) supplemented with 10% fetal bovine serum (FBS) (Omega Scientific), 1% Penicillin/Streptomycin (Gibco), and 2 mM L-glutamine (Gibco). Green fluorescent protein (GFP)-transduced NK92-CD16V cells were provided by Dr Kerry Campbell from Fox Chase Cancer Center, Philadelphia PA. NK92-CD16V cell culture conditions were MEMα (HyClone) supplemented with 10% FBS (Omega Scientific), 10% horse serum (Sigma), 1 mM sodium pyruvate (Gibco), 1X non-essential amino acids (Gibco), 0.2 mM Myo-Inositol (Sigma), and 0.1 mM β-mercaptoethanol (Sigma). NK92-CD16V cells were maintained in suspension and passaged every 2 days by re-suspension at a density of 0.25 × 10^6^ cells/ml and stimulated with 1% v/v of IL-2 supernatant derived from J558L cells as previously described.^21^ All cell lines were maintained at 37°C in 5% CO_2_. Fresh healthy donor NK cells from five different donors were purchased from AllCells with CD56 negative selection (PB012-N). Donor NK cells were expanded and maintained in culture using irradiated K562–4-1BBL-mbIL-21 cells kindly provided by Dr Dean Lee according to his protocol and stimulated with 1% v/v of IL-2 prior to use.^57^ Cell lines were routinely removed from Penicillin/Streptomycin containing medium and tested for mycoplasma every 6 months. Cells were harvested with 0.05% Trypsin-EDTA (ThermoFisher) unless otherwise noted and cell counts for assays were estimated by hemocytometer with viable cells identified by Trypan Blue (Invitrogen) exclusion.

### Derivation of ADCC-Resistant cell lines

Cetuximab (Bristol-Myers Squibb) and trastuzumab (Genentech) were obtained from the MedStar Georgetown University Hospital Pharmacy. A431, SKOV3, and FaDu parental cells were seeded overnight in four *T*-75 flasks at 500,000 cells each. After 24 h, four treatment groups were added: 1) Medium (Untreated); 2) 1 μg/mL cetuximab for A431 and FaDu and 5 μg/mL trastuzumab for SKOV3 (Ab treated); 3) 1,000,000 NK92-CD16V cells for A431 for a 1:1 effector: target ratio and 3,000,000 NK92-CD16V cells for SKOV3 and FaDu for a 3:1 effector: target ratio (NK treated); 4) the combination of Ab and NK treatment for each cell line (ADCC challenge). After 72 h, each flask was aspirated of treatments, washed with HBSS (Cytiva), and the remaining adherent cells were collected and re-seeded to new flasks for subsequent challenge (Ch) with the same treatment conditions. A total of 35 consecutive challenges were conducted for each cell line. After every fifth treatment cycle (Ch5, Ch10, Ch15, etc.) cells from each line from each treatment were expanded for analysis, protein and RNA extraction, and aliquots were cryopreserved in 10% DMSO in FBS at − 80°C.

### Quantification of ADCC sensitivity

Assays to assess the proportion of cells sensitive to ADCC were performed using the Cytotox-Glo Cytotoxicity assay (Promega, G291) in 96-well clear bottom white plates (Corning, 3903) following manufacturer’s protocols. Cells were seeded in wells at 10,000 cells per well overnight. After ~16 hours four treatment groups were added in triplicate: 1) Medium (Target); 2) 1 μg/mL cetuximab or 5 μg/mL trastuzumab for A431 and FaDu and 5 μg/mL trastuzumab or 5 μg/mL cetuximab for SKOV3 (Ab); 3) 20000 NK92-CD16V cells for A431 for 1:1 effector: target ratio and 60,000 NK92-CD16V cells for SKOV3 and FaDu for 3:1 effector: target ratio (Effector) or 100,000 NK cells from a healthy donor for A431, SKOV3, and FaDu for a 5:1 effector: target ratio; 4) the combination of Ab and NK treatment for each cell line (ADCC). Percent cytotoxicity was determined 4-h post exposure to NK92-CD16V or Donor NK cells in the presence or absence of 1 μg/ml of cetuximab or 5 µg/ml of trastuzumab by measuring initial and total cell death as previously described.^20,21^ The percentage of ADCC specific cytotoxicity was calculated as {[(Initial_ADCC_-((Initial_Effector_)+(Initial_Ab_-Initial_Target_))]/[Total_Ab_-Total_background_]}*100.

### Quantification of NK cell conjugation

NK92-CD16V cell conjugation to target cells was assessed using a multi-well conjugation assay. Target cells were plated at a density of 10,000 cells per well on 96-well clear bottom black plates (Greiner 655,090) in FluoroBrite DMEM (Gibco) supplemented with 10% FBS and incubated overnight at 37°C/5% CO_2_. NK92-CD16V cells at a density of 8 × 10^5^cells/ml in Dulbecco’s PBS were labeled with 5 µM carboxyfluorescein diacetate (Molecular Probes) for 20 min at 37°C/5% CO_2_. The labeled NK92-CD16V cells were spun at 1500 rpm for 5 min and resuspended in NK cell medium and incubated for an additional 10 min at 37°C/5% CO_2_. The labeled NK92-CD16V cells were spun again at 1500 rpm for 5 min and resuspended in the FluoroBrite DMEM to 8 × 10^5^cells/ml. NK92-CD16V cells (25 µl representing ~ 1:1 E:T or 3:1 E:T) were added in sextuplets to wells with target cells. Then either 25 µl of medium, cetuximab (1 µg/ml), or trastuzumab (5 µg/ml) was added to target cells. As a background, 50 µl of medium alone was added to a row of target cells. The plate was incubated at 37°C/5% CO_2_ for 2 h and then initial fluorescence was read using PerkinElmer’s Envision 2104 Multilabel Reader set to 492/517 nm excitation/emission. Wells were emptied of non-adhering NK92-CD16V cells, washed twice with 200 µl of FluoroBrite DMEM, refilled with 150 µl FluoroBrite DMEM, and ending fluorescence was measured. Percentage of NK92-CD16V cells in conjugate was calculated as [(fluorescence_end_-fluorescence_background_)/(fluorescence_initial_-fluorescence_background_)]*100. The mean of all replicates for each target cell line was then determined and SEM calculated.

### Crystal violet staining

1.5 × 10^3^ cells from each ADCC-sensitive and – resistant cell line were plated in one well each of 5 6-well plates. At indicated time points, cells were rinsed and subjected to 0.52% Crystal Violet (Fisher Scientific, C581–100) in 25% methanol. For quantitative analysis, cells from each well were dissolved in 100 mmol/L sodium citrate with 50% ethanol and plated in triplicate in 96-well plates. Then, the absorbance was measured using PerkinElmer’s Envision 2104 Multilabel Reader set to 595 nm.

### Flow cytometry

A431, SKOV3, and FaDu cells were collected and resuspended in culture medium to 1 × 10^6^cells/ml. 0.5 × 10^6^ cells were aliquoted into Eppendorf tubes, spun at 5000 rpm for 2 min at 4°C, washed twice with HBSS, and resuspended in 100 μl of FACS buffer (1X PBS (Gibco) + 1% BSA (Sigma)). Labeled antibodies were then added at the manufacturer's recommended concentrations and incubated at 4°C for 30 min. Cells were then washed with FACS buffer twice and resuspended in fixative (1% PFA (Electron Microscopy Sciences) in PBS). Samples were then immediately submitted to the Flow Cytometry & Cell Sorting Shared Resource. Flow antibodies for: EGFR (352906), HER2 (340879), CD54 (322714), APC labeled Mouse IgG1k isotype control (400121), and Alexa Fluor 488 labeled Mouse IgG1k isotype control (400129) were purchased from BioLegend. Samples were run in the Georgetown Lombardi Comprehensive Cancer Center Flow Cytometry & Cell Sorting Shared Resource. Analyses were performed using FlowJo (v10.8.1).

### Cell surface screen

The LEGENDscreen™ Human Cell Surface Marker Screening Panel PE Kit (BioLegend 700,007) containing purified monoclonal antibodies to 361 cell surface markers was employed to map the cell surface proteome. Cells from each ADCC-sensitive and -resistant cell line were collected using Accutase™ (BD Biosciences 561,527) to prevent disruption of cell surface proteins and the assay was conducted according to the manufacturer’s instructions. Samples were run in the Georgetown Lombardi Comprehensive Cancer Center Flow Cytometry & Cell Sorting Shared Resource. The flow cytometry analysis was done using FlowJo (v10.8.1).

### Immunofluorescence assays

Cells were plated in 4-well chamber slides (Nalgene Nunc International) and processed for immunofluorescence microscopy as previously described.^58^ Cetuximab (Bristol-Myers Squibb) and trastuzumab (Genentech) were fluorescently labeled using DyLight® 550 Fast Conjugation Kit (Abcam, ab201800). A431 or FaDu cells were incubated with 1 μg/mL of DyLight® 550 conjugated-cetuximab for 4 h at 37°C and SKOV3 cells were incubated with 5 μg/mL of DyLight® 550 conjugated-trastuzumab for 3 h at 37°C. Following antibody incubation, medium was aspirated and cells were washed in 1X PBS. Cells were immediately fixed by a 20 min incubation with 4% paraformaldehyde at room temperature and an overnight incubation in ice-cold 70% ethanol at 4°C. DAPI staining was done as previously described.^58^ Cells were imaged using Leica SP8.

### Inhibitors and blocking antibodies

The ATR kinase inhibitor VE-821 (Selleckchem, S8007) was dissolved in DMSO to 10 mM. The immunoproteasome inhibitor ONX-0914 (Adooq Bioscience, A12653-10 mM-D) and JAK1/2 kinase inhibitor ruxolitinib (MedChemExpress, HY-50856/C50864) were purchased as 10 mM solutions in DMSO. Prochlorperazine dimaleate salt (Selleckchem, S4631) was dissolved in DMSO to 15 mM. Vehicle treatments (DMSO) were used at the highest equivalent v/v used in inhibitor treatments. Prochlorperazine to a final concentration of 15 μM or vehicle was added 5 min prior to ADCC assays. Cells were incubated with ruxolitinib or vehicle for 72 h and washed prior to plating for downstream ADCC assays. Cells plated for ADCC assays were treated with 1 μM VE-821 or vehicle for 1 hr and then washed prior to ADCC assays. Cells were treated with ONX-0914 for 1 hr prior to ADCC assays. Cells were plated as described in 6-well plates in the presence of ONX-0914 at 3 nM-30 μM concentrations for 24 h prior to crystal violet staining.

Blocking antibodies to CD95 (684401), CD262 (307402), CD104 (327802), CD49f (313602), CD54 (322702) and their related isotype controls: Mouse IgG1-k (400102), Mouse IgG2a-k (400202), Mouse IgG2b-k (400347), and Rat IgG2a-k (400502) were all purchased from BioLegend. An additional replicate of the concomitant block of CD95, CD262, CD104, and CD49f was performed using a blocking antibody to CD95 from ProSpec Bio (ANT-205) and a blocking antibody to CD104 from BD Biosciences (611232) to ensure results were not influenced by ADCC triggered by Mouse IgG2 antibodies. Cells were treated with 10 μg/ml of blocking antibodies or isotype controls for 1 hr prior to ADCC assays.

### RNA extraction and real-time quantitative PCR (RT-qPCR)

RNA was isolated from 1 × 10^6^cells using the PureLink RNA Mini Kit (Ambion 12,183,020). The RNA concentration was measured using NanoDrop 8000 (ThermoFisher Scientific). cDNA was generated from 20 to 100 ng of RNA using the GoTaq 2-step RT-qPCR System (Promega, A6110). RT-qPCR was performed with SYBR Green on a StepOnePlus real-time PCR system (Applied Biosystems). Gene expression in each cell line was normalized to TBP and fold change in expression between ADCC-sensitive and – resistant variants was analyzed using 2^−ΔΔCt^ method.

#### Primer sequences

*ANXA*- (F: TCTACTGTTCACGAAATCCTGTG; R: AGTATAGGCTTTGACAGACCCAT)

*S100A11*- (F: CTGAGCGGTGCATCGAGTC; R: TGTGAAGGCAGCTAGTTCTGTA) *ISG15*- (F: TGGACAAATGCGACGAACCTC; R: TCAGCCGTACCTCGTAGGTG) *IGFBP6*- (F: TGTGAACCGCAGAGACCAAC; R: GCCCATCTCAGTGTCTTGGA)

*FTH1*- (F: TCCTACGTTTACCTGTCCATGT; R: GTTTGTGCAGTTCCAGTAGTGA) *PSMB9*- (F: GGTTCTGATTCCCGAGTGTCT; R: CAGCCAAAACAAGTGGAGGTT)

*EGFR*- (F: AGGCACGAGTAACAAGCTCAC; R: ATGAGGACATAACCAGCCACC) *HER2*- (F: TGCAGGGAAACCTGGAACTC; R: ACAGGGGTGGTATTGTTCAGC)

*TBP*- (F: CCACTCACAGACTCTCACAAC; R: CTGCGGTACAATCCCAGAACT)

### Western bot

Cell pellets of 1 × 10^6^cells were lysed in boiling buffer (Boston BioProducts) supplemented with protease and phosphatase inhibitor (Roche). Lysate protein concentrations were measured by DC Protein Assay (BioRad) following manufacturer protocol. 20 μg lysates were run on SDS-PAGE gels and transferred to nitrocellulose membranes (GE Healthcare). Membranes were blocked for 1 hr in 5% milk (BioRad) in TBST. Western blots were conducted with Cell Signaling antibodies to: E-Cadherin (3195), Vimentin (5741), Snail (3879), Twist (46702), EGFR (4267), pEGFR Y1068 (3777), pEGFR Y1045 (2237), HER2 (4290), pHER2 Y1248 (2247), ERK1/2 (4695), pERK1/2 T202/Y204 (4370), AKT (pan) (4691), pAKT S473 (4060), CD54 (65055), STAT1 (14994), pSTAT1 Y701 (9167), M×1(37849), IL-6 (12153), ATR (2790), pCHK1 S345 (2341), PSMB8 (13635), and PSMB10 (78385) at concentrations of 1:1000 diluted in 5% milk in TBST. Western blots were conducted with Origene antibody to PSMB9 (TA504389) and Abcam antibody to ATM (ab32420) at concentrations of 1:1000 diluted in 5% milk in TBST. Secondary antibodies used were anti-mouse IgG, HRP linked (Cell Signaling, 7076) or anti-rabbit IgG, HRP linked (Cell Signaling, 7074) at concentrations of 1:1000 diluted in 5% milk in TBST. GAPDH (Cell Signaling, 5174) and Alpha Tubulin (Cell Signaling, 2144) antibodies were used at concentrations of 1:10,000 diluted in 5% milk in TBST. The secondary antibody concentration used for GAPDH and Alpha Tubulin was 1:10,000. Chemiluminescent substrate (ThermoFisher, Pico 34,580 or Femto 34,096) was used for visualization. Densitometry was measured using ImageJ (v1.48).

### Reverse phase protein arrays

Protein pathway activation mapping and cell-signaling analysis was performed by reverse-phase protein array (RPPA) as previously described.^59–61^ Cells from each ADCC sensitive and resistance cell line were treated as described, and 12 samples per cell line were processed. Protein signaling analytes were chosen based on their roles in growth factor and proliferation signaling in tumor cells and for any described involvement in the development of resistance to cetuximab or trastuzumab. Detection was performed using a fluorescence-based tyramide signal amplification strategy using Streptavidin-conjugated IRDye680 (LI-COR Biosciences, Lincoln NE) detection reagent. All antibodies were validated for single band specificity and for ligand-induction (for phospho-specific antibodies) by immunoblotting prior to use on the arrays as previously described. Each array was scanned using a TECAN LS (Vidar Systems Corporation, Herndon VA). Spot intensity was analyzed, data were normalized to total protein and a standardized, single data value was generated for each sample on the array by MicroVigene software V2.999 (VigeneTech, North Billerica, MA). For each cell-line pair, unpaired *t*-tests of each analyte were performed and a volcano plot of the Log2 fold change and – Log10(q value) was generated.

### RNA isolation and singe-cell sequencing

Pairs of ADCC-sensitive and -resistant variants of each cell line from challenge 35 were collected and submitted for single-cell sequencing. Single-cell protein and RNA sequencing were performed with cellular indexing of transcriptomes and epitopes by sequencing (scCITE-seq) on each ADCC-sensitive and -resistant A431, FaDu, and SKOV3 cell-line pair. Single-cell RNA labeling and library preparations were performed using the 10X Genomics DyLight® Single-Cell system and DyLight® Single Cell 3’ Library & Gel Bead Kit v3 following the 10X CITE-seq protocol. DyLight® antibodies from BioLegend were added with reactivity for EGFR (352953), HER-2 (324423), CD142/F3 (365207), CD95/FAS (305649), CD49f (313633), CD326/Ep-CAM (324241), CD44 (338825), CD81 (349521), and CD55 (311317). Ten thousand cells were sequenced for each of the six groups. Processing and pseudoalignment were performed with fastqc version 0.11.8, kallisto version 0.46.1, and bustools version 0.39.3. The data were then loaded into R version 4.0.2 using Seurat version 4.0.0. Seurat was used for cell filtering (retaining 20,053 cells), dimensionality reduction with PCA and UMAP, and differential expression analyses of both RNA and protein were performed using a Wilcox test. These data were compared against the signatures of resistance found in the previous A431 data set using the projectR transfer learning package version 1.12.0. Enrichment of GO terms among differentially expressed genes was performed using goSTAG v.1.20.0. To validate the previously identified ADCC resistance signature, transfer learning methods were utilized using ProjectR. The previously published gene signature that defines ADCC resistance^21^ was projected onto the scCITE-seq dataset to correlate known resistance patterns to the multiple cell-line models. Protein–protein networks were determined from STRING analysis of the differentially expressed genes.^62^

To obtain additional temporal resolution of the development of resistance, a single-cell RNAseq data set was also created from populations of A431 cells when sensitive, 24 h post an initial challenge, and when resistance was first observed. Single-cell RNA labeling and library preparations were performed using the 10× Genomics DyLight® Single-Cell system and DyLight® Single Cell 3’ Library & Gel Bead Kit v2 (10× Genomics), following the manufacturer’s instructions. An input of 8700 was used to recover a total of 5,000 cells. Sequencing was performed using the HiSeq platform (Illumina) for 2 × 100bp sequencing and ~50,000 reads per cell. Samples were sequenced in duplicate. Sequences were filtered and aligned using the CellRanger software (10× Genomics). Single-cell analysis was performed utilizing Monocle (Version 2.10.1), and sequencing data were filtered to remove low-quality cells, samples with outlier mRNA density to remove potential doublets or triplets, and dimensionality was reduced using principal component analysis. Genes expressed within the dataset were required to be expressed in at least 10 cells, and gene expression was log transformed. Pseudo-time analysis was also performed using Monocle by clustering expressed genes by treatment classification, and gene expression changes were analyzed as a function of pseudo-time.

### Single-cell ATAC sequencing and analysis

Concurrent with scCITE-seq, single-cell assay for transposase-accessible chromatin using sequencing (scATAC-seq) was performed on the same cell lines. Nuclei were isolated, and library preparation was performed using Chromium Single-Cell ATAC Reagent kit v1.1. Processing of the data was performed using Cell Ranger ATAC version 2.1. The data were then analyzed using ArchR version 1.0.1^63^ to filter low transcriptional start site enrichment or low fragment cells and remove potential doublets. ArchR was then used for dimensionality reduction using Latent Semantic Indexing and UMAP as well as differential accessibility testing.

### siRNA and CD54 transfection

Gene-specific siRNAs for STAT1 (Cell Signaling, 6331) or nonspecific scramble control siRNA (siNEG, Qiagen 1,027,281) were used. 12.5 nM of siRNAs were reversely transfected to cells with RNAiMax transfection reagent (Invitrogen 13,778,075) for 48 h according to manufacturer’s instructions. Then cells were harvested for protein and plated for downstream ADCC assays. Transfection of CD54 (ICAM1) in A431 cells was carried out by lipofection using Lipofectamine 3000™ (ThermoFisher), according to the manufacturer’s instructions. In brief, 5 × 10^5^A431 cells were seeded in 2 ml of complete medium and after 24 h transfection was performed with 2 μg of pICAM1, a full-length ICAM1 cDNA inserted in the CDM8 expression vector (a gift from Timothy Springer [Addgene plasmid #8632; http://n2t.net/addgene:8632; RRID:Addgene_8632]).

### CRISPR/Cas9 knockout

STAT1 knockout (KO) and non-targeting control (NTC) cell lines were generated with the Edit-R Gene Editing System (Horizon). Two STAT1 Edit-R crRNA (CM-003543-01, CM-003543-02) were used singly or together with Edit-R Synthetic tracrRNA (U-002005). One NTC was generated (U-007501). Transfections were set up with 7000 cells/well, 50 nM crRNA:tracrRNA, 25 nM Cas9 Nuclease Protein NLS (CAS11200) and 0.35 µg/well of DharmaFECT Duo transfection reagent (*T*-2010). Cells were harvested after 3 days and single-cell-sorted into 96-well plates; multiple clones were generated and compared for STAT1 expression by RT-qPCR and western blot, and assessed for their proliferative rate as compared to parental A431 cells. The presented data are of one selected NTC and STAT1 KO clone.

### Immunoproteasome activity

1 × 10^6^ cells from each cell line were collected as described. Immunoproteasome activity was assessed using the 20S Immunoproteasome Activity Assay Kit (BioVision, K2051) in 96-well clear bottom white plates (Corning, 3903) following the manufacturer protocol. In brief, cells were lysed in assay buffer and lysate was plated. In some wells, cells were treated with either 30 nM or 300 nM ONX-0914 (Adooq Bioscience, A12653-10 mM-D). Plates were mixed and incubated at 37°C for 1 h. Fluorescence readings were taken at 5-min intervals using an excitation wavelength of 345 nm and an emission of 445 nm. Relative fluorescence units (RFU) were calculated with reference to an AMC standard curve of known amounts of AMC following subtraction of background fluorescence.

### Statistical analysis

Data analysis involved use of Prism software (GraphPad). Results are expressed as mean±S.E.M. Differences were analyzed by student’s *t*-test, and results were considered significant at *P* < .05.

## Supplementary Material

Supplemental Material

Supplemental Material

Supplemental Material

## Data Availability

The data that support the findings of this study are in GEO, accession number GSE206955 and are available upon request from the corresponding author, LMW.
