# Supplementary material for: Antibody dependent cell-mediated cytotoxicity selection pressure induces diverse mechanisms of resistance
Source: Cancer Biol Ther. 2023 Oct 25;24(1):2269637. doi: 10.1080/15384047.2023.2269637 (PMC10601508; doi:10.1080/15384047.2023.2269637)
Supplement: Supplemental Material [file KCBT_A_2269637_SM9689.docx]

**Supplementary Table 2: LEGENDScreen Geometric Mean Values in ADCC-Sensitive (ADCCS) and ADCC-Resistant (ADCCR) Cell Lines**

|  | **A431**  **ADCCS** | **A431**  **ADCCR** | **SKOV3 ADCCS** | **SKOV3 ADCCR** | **FaDu**  **ADCCS** | **FaDu**  **ADCCR** |
| --- | --- | --- | --- | --- | --- | --- |
| No Ab Ctrl | 3.59 | 3.7 | 5.59 | 6.95 | 6.42 | 3.92 |
| Armenian Hamster IgG Isotype Ctrl | 4.07 | 3.98 | 6.25 | 8.31 | 6.51 | 3.91 |
| CCR10 | 18.1 | 11.3 | 20.3 | 38.9 | 10 | 5.45 |
| CD278 (ICOS) | 4.2 | 3.83 | 6.16 | 7.94 | 6.42 | 3.77 |
| IFN-yRB Chain | 4.25 | 4.01 | 6.26 | 8.12 | 6.85 | 4.22 |
| Mouse IgG1, k Isotype Ctrl | 4.54 | 4.15 | 6.16 | 8.78 | 6.76 | 4.15 |
| CD46 | 52.7 | 41.1 | 110 | 131 | 60.4 | 42.1 |
| CD70 | 4.3 | 3.91 | 68.6 | 183 | 6.79 | 4.12 |
| CD1a | 4.06 | 3.69 | 6.14 | 8.37 | 6.6 | 3.9 |
| CD2 | 3.64 | 3.15 | 5.13 | 6.6 | 6.2 | 3.83 |
| B2 Microglobulin | 2934 | 4860 | 6585 | 8081 | 2015 | 2644 |
| B7-H4 | 4.19 | 8.57 | 6.02 | 6.83 | 6.5 | 3.97 |
| Cadherin 11 | 4.54 | 3.81 | 6.18 | 7.24 | 6.81 | 4.04 |
| CD10 | 3.86 | 3.52 | 7.78 | 42.1 | 12.9 | 7.31 |
| CD100 | 5.41 | 4.4 | 7.42 | 9.02 | 7.33 | 4.32 |
| CD103 | 3.53 | 2.92 | 5.48 | 7.2 | 6 | 3.79 |
| CD105 | 7.14 | 3.4 | 5.8 | 7.05 | 103 | 68.2 |
| CD106 | 3.73 | 3.14 | 5.53 | 6.9 | 6.87 | 5.07 |
| CD107a (LAMP-1) | 25.4 | 33.8 | 64.9 | 155 | 89.1 | 97.8 |
| CD107b (LAMP-2) | 18.3 | 25.9 | 42.9 | 61.6 | 74.6 | 43 |
| CD109 | 15.4 | 12.7 | 10.9 | 21.1 | 25.5 | 23.3 |
| CD111 | 23.8 | 37.2 | 16.3 | 26.8 | 289 | 197 |
| CD112 (Nectin-2) | 134 | 126 | 193 | 230 | 95.2 | 85 |
| CD114 | 5.73 | 3.29 | 5.87 | 7.41 | 6.33 | 3.99 |
| CD116 | 4.02 | 3.31 | 6.11 | 7.39 | 6.9 | 4.42 |
| CD117 (c-kit) | 3.91 | 3.29 | 6.42 | 29.4 | 6.17 | 3.92 |
| CD119 (IFN-y R a Chain) | 13.3 | 13.9 | 20.5 | 21.5 | 18.8 | 14.3 |
| CD11a | 3.73 | 2.96 | 5.58 | 6.89 | 6.22 | 5.18 |
| CD11b | 3.81 | 3.09 | 5.85 | 7.14 | 6.26 | 3.77 |
| CD122 | 3.61 | 2.99 | 16.4 | 6.82 | 6.08 | 3.69 |
| CD123 | 3.87 | 3.12 | 5.1 | 8.2 | 6.04 | 3.7 |
| CD126 (IL-6Ra) | 5.39 | 4.26 | 7.46 | 12.2 | 6.9 | 4.56 |
| CD127 (IL-7Ra) | 3.87 | 3.11 | 5.19 | 10.3 | 6.51 | 4.38 |
| CD13 | 4.34 | 3.68 | 34.2 | 981 | 6.57 | 3.82 |
| CD131 | 3.98 | 3.2 | 8.59 | 7.86 | 5.88 | 3.68 |
| CD134 | 4.03 | 2.96 | 9.88 | 8.36 | 6.98 | 3.8 |
| CD135 | 4.54 | 3.25 | 5.85 | 7.18 | 6.35 | 4.2 |
| CD137 (4-1BB) | 4.08 | 3.63 | 5.8 | 7.03 | 6.16 | 3.67 |
| CD137L (4-1BB Ligand) | 16.3 | 12.3 | 17.3 | 20.5 | 12.6 | 12 |
| CD138 | 199 | 220 | 38.7 | 28.9 | 98 | 63.9 |
| CD14 | 4.44 | 4.67 | 6.28 | 8.49 | 13.5 | 9.91 |
| CD140a | 3.92 | 3.3 | 6.23 | 7.13 | 6.15 | 3.87 |
| CD140b | 4.64 | 4.11 | 6.68 | 11.3 | 6.43 | 4.24 |
| CD141 | 228 | 63.3 | 36.9 | 51.4 | 90.8 | 66.4 |
| CD142 | 2883 | 895 | 175 | 1838 | 9464 | 1763 |
| CD143 | 4.19 | 4.33 | 5.95 | 8.36 | 6.16 | 3.89 |
| CD146 | 3.78 | 3.35 | 12.3 | 17.7 | 6.62 | 4.16 |
| CD148 | 8.72 | 11.3 | 92.4 | 54.7 | 6.54 | 4.32 |
| CD15 | 15.3 | 5.73 | 5.71 | 6.81 | 15.1 | 16.4 |
| CD150 (SLAM) | 3.7 | 2.98 | 7.58 | 6.8 | 6 | 3.81 |
| CD151 | 136 | 171 | 932 | 797 | 206 | 133 |
| CD154 | 3.94 | 3.23 | 6.19 | 6.82 | 5.83 | 3.69 |
| CD156c (ADAM10) | 168 | 140 | 619 | 567 | 631 | 632 |
| CD158e1 | 3.72 | 3.54 | 5.73 | 7.55 | 5.96 | 4.9 |
| CD16 | 3.8 | 3.33 | 7 | 6.83 | 5.97 | 3.98 |
| CD161 | 3.9 | 5.13 | 7.8 | 6.96 | 5.72 | 3.58 |
| CD162 | 3.84 | 3.38 | 5.93 | 8.74 | 5.86 | 3.62 |
| CD163 | 4.33 | 3.64 | 6.82 | 7.45 | 5.92 | 3.9 |
| CD164 | 225 | 187 | 253 | 1091 | 655 | 191 |
| CD165 | 3.93 | 2.95 | 5.9 | 6.6 | 5.87 | 3.57 |
| CD166 | 378 | 343 | 1660 | 2295 | 1383 | 324 |
| CD169 | 3.59 | 3.88 | 5.42 | 6.66 | 5.99 | 5.42 |
| CD170 (Siglec-5) | 70.7 | 74.5 | 57.3 | 45.5 | 102 | 63.7 |
| CD172a/b (SIRPa/b) | 14.7 | 15.4 | 11.5 | 87.4 | 6.91 | 4.68 |
| CD172g (SIRPy) | 10 | 9.8 | 9.05 | 46.6 | 6.21 | 3.65 |
| CD178 (Fas-L) | 4.36 | 3.64 | 5.85 | 7.06 | 6.09 | 3.77 |
| CD179a | 4.9 | 4.03 | 6.79 | 8.88 | 7.01 | 3.89 |
| CD179b | 3.91 | 3.08 | 5.44 | 6.93 | 6.04 | 3.67 |
| CD18 | 4.08 | 3.18 | 5.85 | 6.79 | 6.18 | 3.84 |
| CD180 (RP105) | 3.8 | 2.92 | 5.38 | 6.73 | 5.94 | 3.64 |
| CD182 (CXCR2) | 3.73 | 2.94 | 5.22 | 6.19 | 6.07 | 3.94 |
| CD183 | 4.88 | 3.51 | 7.23 | 7.55 | 6.29 | 3.9 |
| CD185 | 4.57 | 3.63 | 6.52 | 7.51 | 7.87 | 3.99 |
| CD19 | 3.77 | 2.95 | 5.46 | 6.81 | 7.47 | 12.1 |
| CD191 | 4.14 | 3.38 | 9.35 | 14.5 | 6.08 | 4.09 |
| CD194 | 3.99 | 3.1 | 5.74 | 7.03 | 6.22 | 3.91 |
| CD1b | 3.96 | 3.23 | 6.59 | 7.5 | 6.33 | 3.94 |
| CD1c | 3.7 | 2.95 | 5.4 | 6.27 | 5.84 | 3.63 |
| CD200 | 4.2 | 3.33 | 12.7 | 7.41 | 5.98 | 3.76 |
| CD200R | 4.82 | 3.09 | 6.1 | 7.34 | 5.95 | 3.78 |
| CD202b | 4.32 | 3.26 | 5.34 | 6.82 | 6 | 3.5 |
| CD203c | 10.3 | 8.03 | 10.2 | 12.4 | 6.73 | 3.91 |
| CD205 | 9.86 | 11 | 19.5 | 8.31 | 5.78 | 3.44 |
| CD206 | 3.9 | 3.05 | 5.35 | 6.64 | 6.01 | 3.38 |
| CD207 | 3.8 | 3.13 | 5.68 | 6.47 | 6.19 | 3.53 |
| CD21 | 3.51 | 2.86 | 5.14 | 6.36 | 5.91 | 3.39 |
| CD213a1 | 5.02 | 5.19 | 10.4 | 10.3 | 6.12 | 3.77 |
| CD213a2 | 3.71 | 2.91 | 5.82 | 6.57 | 6.12 | 3.83 |
| CD218a (IL-18Ra) | 4.25 | 3.74 | 6.86 | 7.54 | 9.2 | 5.06 |
| CD221 (IGF-1R) | 23.3 | 22.2 | 14.4 | 26.1 | 34.1 | 21 |
| CD223 | 3.8 | 2.85 | 5.06 | 6.49 | 5.8 | 3.39 |
| CD226 | 4.19 | 3.37 | 5.5 | 6.68 | 5.91 | 3.49 |
| CD227 | 7.04 | 22.8 | 79 | 76.9 | 20.5 | 212 |
| CD229 (Ly-9) | 3.61 | 2.7 | 5.24 | 5.93 | 5.79 | 3.65 |
| CD23 | 3.57 | 2.58 | 5.08 | 5.76 | 5.59 | 3.38 |
| CD231 | 4.27 | 2.99 | 5.67 | 7.14 | 6 | 3.5 |
| CD244 | 3.7 | 2.89 | 5.91 | 8.31 | 5.7 | 3.29 |
| CD245 | 6.63 | 4.61 | 7.2 | 8.93 | 7.42 | 3.99 |
| CD25 | 3.82 | 5.36 | 4.89 | 6.91 | 6.45 | 3.31 |
| CD252 (OX40L) | 14.9 | 17.4 | 26.2 | 31 | 8.69 | 4.8 |
| CD261 (DR4, TRAIL-R1) | 5.08 | 3.62 | 9.85 | 9.19 | 6.01 | 3.6 |
| CD262 (DR5, TRAIL-R2) | 36.1 | 12.1 | 56.4 | 36.8 | 25.6 | 13.9 |
| CD263 | 5.27 | 6.65 | 7.45 | 7.2 | 6.18 | 4.74 |
| CD266 (Fn14, TWEAK Receptor) | 147 | 24.6 | 117 | 152 | 170 | 42.7 |
| CD268 | 3.78 | 2.72 | 4.96 | 6.18 | 5.51 | 3.2 |
| CD278 (ICOS) | 3.97 | 2.78 | 4.96 | 6.66 | 5.72 | 3.31 |
| CD271 | 48.8 | 25.6 | 5.74 | 6.19 | 108 | 44.6 |
| CD275 (ICOS Ligand) | 4.78 | 4.26 | 13.2 | 9.52 | 10.9 | 5.76 |
| CD276 (B7-H3) | 141 | 92.5 | 960 | 1254 | 686 | 408 |
| CD277 | 7157 | 3135 | 8654 | 8179 | 8747 | 5070 |
| CD279 (PD-1) | 3.69 | 3.69 | 5.53 | 7.15 | 5.8 | 3.24 |
| CD28 | 3.84 | 3.63 | 6.15 | 7.32 | 5.86 | 4.83 |
| CD29 | 1492 | 1555 | 6285 | 9130 | 2797 | 1470 |
| CD290 | 4.09 | 4.42 | 6.43 | 7.2 | 5.93 | 3.65 |
| CD298 | 4289 | 3035 | 2325 | 1508 | 6050 | 3047 |
| CD3 | 3.72 | 3.18 | 5.65 | 7.29 | 5.72 | 3.25 |
| CD30 | 3.76 | 3.46 | 5.23 | 6.52 | 5.91 | 3.14 |
| CD300c | 5.05 | 3.58 | 6.71 | 9.47 | 5.94 | 3.4 |
| CD309/VEGFR2 | 3.97 | 3.26 | 5.24 | 7.18 | 5.98 | 3.37 |
| CD31 | 3.8 | 3.04 | 5.68 | 6.4 | 5.82 | 3.25 |
| CD314 | 4.17 | 3.23 | 5.76 | 7.81 | 5.9 | 3.28 |
| CD317 | 95.6 | 37.1 | 200 | 83.3 | 6.92 | 8.18 |
| CD324 (E-Cadherin) | 123 | 119 | 81.6 | 33.3 | 252 | 362 |
| CD325 | 4.33 | 3.54 | 9.49 | 11.4 | 6.4 | 3.59 |
| CD328 | 3.69 | 2.83 | 171 | 6.23 | 5.57 | 3.23 |
| CD33 | 5.05 | 3.47 | 15.5 | 7.21 | 5.86 | 3.46 |
| CD334 | 3.82 | 2.98 | 6.16 | 6.59 | 5.6 | 3.21 |
| CD335 | 3.67 | 2.88 | 5.44 | 6.11 | 5.54 | 3.25 |
| CD336 | 3.82 | 3.2 | 5.14 | 6.17 | 6.53 | 3.04 |
| CD337 | 3.96 | 3.2 | 5.09 | 7.36 | 5.57 | 3.06 |
| CD34 | 4.39 | 2.9 | 4.97 | 6.03 | 5.4 | 3.08 |
| CD340 (HER2) | 49.5 | 61.6 | 7225 | 609 | 75 | 78.7 |
| CD344 | 4.98 | 4.98 | 12.8 | 11.8 | 6.71 | 4.09 |
| CD35 | 3.63 | 3.32 | 5.32 | 5.91 | 5.51 | 3.18 |
| CD354 | 5.27 | 3.5 | 5.57 | 6.82 | 5.88 | 3.5 |
| CD360 | 4.46 | 3.55 | 5.57 | 7.01 | 5.95 | 3.55 |
| CD365 | 5.9 | 5.19 | 17.6 | 8.24 | 9.41 | 5.87 |
| CD366 | 3.9 | 3.61 | 5.64 | 7.28 | 5.68 | 3.35 |
| CD367 | 6.04 | 3.56 | 5.58 | 7 | 6.45 | 3.35 |
| CD36L1 | 89.5 | 36.8 | 53.3 | 62.6 | 80.4 | 112 |
| CD38 | 10.1 | 3.7 | 7.5 | 15.3 | 5.75 | 3.32 |
| CD39 | 4.18 | 3.74 | 5.38 | 737 | 6.76 | 3.76 |
| CD46 | 3.97 | 3.74 | 5.04 | 11.5 | 5.39 | 3.14 |
| CD40 | 35.2 | 46.8 | 6.2 | 24 | 5.39 | 3.71 |
| CD41 | 3.51 | 2.83 | 5.12 | 6.09 | 5.31 | 3.05 |
| CD42b | 3.79 | 2.99 | 5.64 | 7.04 | 5.38 | 3.03 |
| CD43 | 5.56 | 4.8 | 9.66 | 12.7 | 6.81 | 3.79 |
| CD44 | 113 | 46.2 | 883 | 681 | 168 | 9 |
| CD45 | 3.74 | 3 | 5.32 | 7.09 | 5.45 | 3.04 |
| CD47 | 436 | 651 | 970 | 805 | 902 | 417 |
| CD48 | 4.3 | 3.55 | 6.98 | 8.99 | 5.71 | 3.16 |
| CD49a | 10.5 | 11.2 | 146 | 362 | 14.3 | 4.35 |
| CD49b | 137 | 139 | 58.1 | 78.5 | 366 | 146 |
| CD49c | 844 | 910 | 3585 | 7363 | 1466 | 779 |
| CD49d | 6.2 | 3.1 | 17.8 | 41.1 | 5.53 | 3.09 |
| CD5 | 3.61 | 2.86 | 5.42 | 6.55 | 5.48 | 3.16 |
| CD50 | 3.63 | 2.8 | 5.2 | 7.61 | 5.32 | 3.08 |
| CD54 | 58.7 | 24.8 | 7.33 | 12.7 | 31.8 | 39.2 |
| CD55 | 79.7 | 51.8 | 131 | 154 | 321 | 59.4 |
| CD56 | 4.63 | 2.98 | 5.9 | 6.85 | 6.24 | 3.44 |
| CD58 | 515 | 338 | 373 | 340 | 649 | 475 |
| CD6 | 4.39 | 2.85 | 5.36 | 8.58 | 7.19 | 3.05 |
| CD61 | 4.03 | 3.24 | 150 | 322 | 5.51 | 4.78 |
| CD62E | 4.44 | 3.43 | 6.19 | 7.14 | 5.68 | 3.43 |
| CD62L | 4.75 | 3.44 | 5.95 | 6.99 | 5.55 | 3.32 |
| CD62P | 5 | 3.08 | 6.11 | 152 | 5.63 | 3.12 |
| CD63 | 308 | 154 | 779 | 1453 | 408 | 159 |
| CD64 | 4.11 | 3.17 | 5.84 | 9.56 | 5.83 | 3.35 |
| CD69 | 3.71 | 2.86 | 13.4 | 5.97 | 5.44 | 3.1 |
| CD73 | 336 | 114 | 234 | 726 | 90.6 | 14 |
| CD74 | 6.07 | 4.3 | 8.29 | 8.04 | 6.18 | 3.49 |
| CD79b | 4.2 | 3.2 | 6.07 | 6.9 | 6.09 | 3.45 |
| CD8 | 3.97 | 2.96 | 5.16 | 5.54 | 5.49 | 3.3 |
| CD80 | 4.8 | 3.26 | 6.01 | 7.59 | 5.9 | 3.32 |
| CD81 | 1433 | 924 | 1799 | 2298 | 462 | 83.1 |
| CD82 | 181 | 383 | 19.4 | 59.5 | 182 | 268 |
| CD83 | 28 | 3.91 | 6.11 | 7.74 | 6.37 | 3.5 |
| CD85g | 4.04 | 3.34 | 7.16 | 12.2 | 5.7 | 3.25 |
| CD85k | 3.97 | 4.48 | 5.98 | 7.41 | 5.73 | 3.06 |
| CD87 | 4.72 | 3.44 | 6.41 | 7.45 | 5.96 | 3.74 |
| CD89 | 4.25 | 3.08 | 5.58 | 6.97 | 5.75 | 3.3 |
| CD8a | 3.82 | 2.85 | 5.1 | 6.42 | 5.51 | 2.99 |
| CD9 | 2909 | 3213 | 2969 | 4118 | 6121 | 6546 |
| CD90 | 3.52 | 2.86 | 5.52 | 6.03 | 5.59 | 3.15 |
| CD93 | 4.61 | 4.06 | 6.53 | 7.14 | 5.6 | 3.18 |
| CD94 | 3.57 | 2.75 | 5.14 | 5.82 | 6.08 | 3.01 |
| CD95 | 208 | 119 | 99.8 | 40.4 | 175 | 126 |
| CD96 | 6.3 | 3.2 | 5.6 | 7.61 | 6.46 | 3.25 |
| CD97 | 26.8 | 20.9 | 36.8 | 15.2 | 6.8 | 4.66 |
| CD99 | 167 | 246 | 146 | 381 | 205 | 215 |
| CXCL16 | 3.92 | 3.45 | 5.8 | 8.77 | 5.8 | 3.31 |
| DLL1 | 3.98 | 3.22 | 4.61 | 5.88 | 9.56 | 3.58 |
| DLL4 | 3.56 | 2.92 | 4.79 | 5.82 | 5.36 | 2.9 |
| DR3 | 4.09 | 3 | 5.46 | 7.16 | 6.21 | 3.38 |
| EGFR | 9585 | 1037 | 442 | 673 | 2076 | 631 |
| GITR | 4.22 | 3.16 | 5.45 | 9.5 | 16.3 | 3.09 |
| GPR19 | 6.15 | 6.12 | 46.8 | 28.7 | 13.1 | 7.7 |
| GPR56 | 18.3 | 37.8 | 14.2 | 23.3 | 12 | 8.68 |
| HLA-E | 4.35 | 5.97 | 5.69 | 7.14 | 6.38 | 3.87 |
| HVEM | 3.46 | 2.53 | 23.2 | 5.54 | 5.71 | 2.92 |
| Ig Light Chain K | 3.45 | 3.03 | 4.68 | 5.51 | 5.33 | 2.87 |
| IgM | 3.79 | 2.97 | 5.34 | 6.11 | 5.84 | 2.98 |
| IL-21R | 4.27 | 3.1 | 6 | 7.13 | 5.75 | 3.06 |
| Integrin a9B1 | 4.27 | 3.51 | 6.22 | 9.42 | 10.8 | 3.5 |
| Jagged 2 | 3.67 | 2.66 | 4.77 | 6.44 | 5.22 | 2.88 |
| Ksp37 | 4.1 | 3.03 | 5.43 | 7.04 | 5.51 | 2.91 |
| LAP | 4.03 | 2.98 | 5.13 | 6.7 | 7.31 | 2.88 |
| LY6G6D | 13.9 | 9.25 | 9.11 | 8.52 | 5.78 | 3.41 |
| MERTK | 45.9 | 51.9 | 33.5 | 29.6 | 183 | 118 |
| MSC (W7C6) | 13.9 | 6.71 | 12.5 | 13.9 | 14 | 6.93 |
| MSC and NPC (W4A5) | 3.52 | 2.76 | 4.98 | 6.21 | 5.6 | 2.89 |
| MSCA-1 | 3.81 | 3.02 | 5.7 | 7.48 | 5.81 | 3.74 |
| MUC-13 | 4.26 | 4.26 | 8.76 | 8.91 | 6.09 | 3.17 |
| NKp80 | 4.44 | 3.15 | 6.48 | 7.32 | 6.82 | 3.45 |
| Notch 1 | 3.95 | 3.87 | 4.68 | 6.3 | 6 | 3.91 |
| Notch 3 | 7.52 | 4.69 | 10.9 | 15 | 7.43 | 3.71 |
| Notch 4 | 8.18 | 5.42 | 10.3 | 13.7 | 9.31 | 4.84 |
| NPC | 3.92 | 2.84 | 7.47 | 6.86 | 5.68 | 34.9 |
| NTB-A | 3.75 | 2.56 | 5.22 | 7 | 5.94 | 3.27 |
| PSMA | 21.2 | 13.9 | 12.3 | 9.27 | 6.65 | 3.92 |
| ROR1 | 4.91 | 3.08 | 5.72 | 7.47 | 7.21 | 2.92 |
| Siglec-10 | 4.17 | 3 | 5.5 | 7.17 | 5.79 | 2.99 |
| Siglec-7 | 3.86 | 3.65 | 5.48 | 6.67 | 5.74 | 2.85 |
| Siglec-8 | 3.46 | 3.67 | 5.3 | 6.17 | 5.22 | 2.74 |
| Siglec-9 | 4.84 | 3.18 | 17.9 | 9.06 | 5.51 | 2.76 |
| SSEA-5 | 5.72 | 20.6 | 45.6 | 8.15 | 9.86 | 4.09 |
| SUSD2 | 986 | 1025 | 504 | 35.6 | 2425 | 1615 |
| TCR a/B | 6.05 | 4.22 | 7.16 | 8.99 | 6.2 | 3.38 |
| TCR g/d | 4.39 | 3.68 | 5.24 | 7.01 | 5.4 | 3.03 |
| Tim-4 | 4.75 | 3.15 | 5.07 | 6.73 | 5.47 | 2.9 |
| TLT-2 | 3.6 | 2.73 | 4.83 | 8.54 | 5.15 | 2.73 |
| TM4SF20 | 4.04 | 3.34 | 7.55 | 9.86 | 5.79 | 3.26 |
| TRA-2-49 | 3.78 | 2.8 | 5.52 | 7.1 | 5.51 | 2.94 |
| TRA-2-54 | 4.57 | 3.09 | 4.93 | 6.72 | 5.71 | 3.17 |
| TSLPR | 3.83 | 2.98 | 5.6 | 6.95 | 5.61 | 2.97 |
| VEGFR3 | 4.06 | 2.85 | 5.69 | 6.99 | 5.63 | 3.04 |
| Mouse IgG2a, k Isotype Ctrl | 3.86 | 2.62 | 4.78 | 6.79 | 5.81 | 2.93 |
| APCDD1 | 4 | 2.89 | 5.16 | 6.76 | 5.79 | 3.22 |
| BTLA | 4.35 | 3.26 | 6.03 | 7.11 | 6.01 | 3 |
| CCR8 | 6.86 | 5.53 | 11.5 | 16.1 | 6 | 3.37 |
| CCRL2 | 5.62 | 6.89 | 7.85 | 8.96 | 6.25 | 3.56 |
| CD102 | 3.82 | 2.67 | 9.72 | 6.74 | 5.31 | 2.92 |
| CD104 | 196 | 105 | 132 | 27.2 | 134 | 19.2 |
| CD124 | 6.81 | 4.93 | 9.11 | 8.91 | 7.01 | 3.64 |
| CD130 | 7.65 | 10.2 | 16.8 | 37.1 | 9.32 | 4.07 |
| CD144 | 4.4 | 3.26 | 20.6 | 8.49 | 6.1 | 3.33 |
| CD152 | 5.06 | 3.39 | 5.71 | 8.56 | 5.56 | 2.9 |
| CD155 | 291 | 237 | 623 | 712 | 331 | 204 |
| CD158b | 4.29 | 2.96 | 5.24 | 8.21 | 5.58 | 2.93 |
| CD184 | 4.68 | 3.78 | 7.21 | 11.7 | 6.62 | 3.13 |
| CD186 | 4.16 | 3.04 | 5.88 | 9.13 | 5.3 | 2.88 |
| CD192 | 4.38 | 3.66 | 7.8 | 9.23 | 6.51 | 3.76 |
| CD197 | 4.33 | 3.49 | 13.5 | 14 | 6.08 | 3.25 |
| CD199 | 5.38 | 4 | 5.52 | 7.81 | 6.17 | 3.25 |
| CD209 | 3.64 | 2.73 | 4.87 | 6.43 | 5.53 | 2.83 |
| CD217 | 8.96 | 7.49 | 12.4 | 21.4 | 12.3 | 7.27 |
| CD230 | 10.6 | 8.98 | 16.3 | 16.3 | 21.1 | 13 |
| CD24 | 4.15 | 4.4 | 631 | 381 | 5.81 | 2.99 |
| CD243 | 4.1 | 3.26 | 5.95 | 9.37 | 9.42 | 3.95 |
| CD26 | 3.76 | 2.98 | 1155 | 103 | 5.41 | 2.82 |
| CD269 | 3.75 | 3.33 | 5.35 | 6.68 | 5.53 | 2.71 |
| CD282 | 4.06 | 3.58 | 6.17 | 7.35 | 5.76 | 3.22 |
| CD284 | 4.85 | 4.42 | 6.33 | 8.52 | 6.45 | 3.02 |
| CD301 | 6.83 | 5.67 | 7.19 | 8.93 | 5.91 | 4.5 |
| CD303 | 3.81 | 3.16 | 4.97 | 6.35 | 5.37 | 2.83 |
| CD304 | 4.88 | 3.39 | 17.6 | 19.8 | 7.53 | 4.19 |
| CD307 | 3.54 | 2.62 | 4.79 | 6 | 5.4 | 2.79 |
| CD323 | 3.57 | 3.95 | 4.81 | 6.04 | 5.69 | 2.81 |
| CD357 | 3.58 | 2.9 | 4.72 | 5.96 | 5.6 | 2.9 |
| CD36 | 3.73 | 3.13 | 4.92 | 7.67 | 5.81 | 2.98 |
| CD369 | 3.95 | 3.29 | 5.78 | 7.05 | 5.87 | 3.71 |
| CD370 | 9.85 | 2.9 | 5.78 | 6.43 | 5.86 | 2.86 |
| CD371 | 3.39 | 2.63 | 4.51 | 5.76 | 5.18 | 2.92 |
| CD45RO | 3.67 | 2.73 | 4.85 | 5.75 | 5.3 | 2.68 |
| CD51 | 87.5 | 106 | 793 | 1312 | 50.7 | 39.5 |
| CD59 | 976 | 1063 | 3719 | 4067 | 4641 | 1639 |
| CD7 | 3.86 | 3.07 | 4.81 | 6.27 | 5.48 | 2.73 |
| CD71 | 542 | 725 | 1538 | 1584 | 2242 | 898 |
| CD84 | 4.7 | 4.01 | 8.55 | 6.93 | 9.01 | 3.38 |
| CD88 | 3.91 | 3.04 | 5.09 | 6.35 | 5.55 | 3.08 |
| CRTAM | 5.24 | 3.32 | 5.52 | 6.53 | 5.55 | 2.95 |
| HER-3 | 17.9 | 19.2 | 5.44 | 7.27 | 25.8 | 32.6 |
| FPR3 | 4.26 | 3.21 | 6.14 | 6.9 | 5.9 | 2.92 |
| Ganglioside GD2 | 3.78 | 3.14 | 5.52 | 6.26 | 5.38 | 2.72 |
| GPR83 | 4.91 | 3.14 | 6.01 | 8.22 | 6.39 | 3.01 |
| HLA-A,B,C | 362 | 638 | 902 | 1063 | 422 | 417 |
| HLA-DR | 4.41 | 6.62 | 4.82 | 5.74 | 6.34 | 6.42 |
| Ig Light Chain λ | 3.68 | 2.78 | 9.46 | 8.35 | 4.9 | 2.89 |
| IgD | 4.17 | 3.14 | 5.6 | 6.64 | 5.4 | 3.09 |
| IL-28RA | 3.71 | 2.99 | 5.08 | 6.24 | 5.37 | 2.89 |
| Integrin b5 | 19.5 | 19.1 | 44.5 | 29.1 | 11 | 6.61 |
| KLRG1 | 4.2 | 3.12 | 5.4 | 6.75 | 5.6 | 3.66 |
| LOX-1 | 4.82 | 13.8 | 5.14 | 6.37 | 5.26 | 5.73 |
| MICA/MICB | 5.21 | 4.14 | 5.17 | 6.49 | 20.1 | 8.11 |
| MSC | 5.54 | 21.9 | 46.7 | 7.65 | 9.7 | 5.01 |
| Notch 2 | 6.43 | 4.85 | 6.2 | 7.71 | 7.01 | 3.34 |
| TACSTD2 | 275 | 706 | 976 | 270 | 2743 | 3352 |
| TIGIT | 3.55 | 2.68 | 4.76 | 6.01 | 4.87 | 2.86 |
| Mouse IgG2b, k Isotype Ctrl | 4.61 | 3.13 | 5 | 6.7 | 5.54 | 3.36 |
| C3AR | 3.9 | 2.9 | 5.32 | 6.37 | 5.37 | 3.02 |
| CCX-CKR | 4.8 | 3.13 | 7.19 | 11.2 | 9.1 | 3.46 |
| CD11c | 3.77 | 3.29 | 5.68 | 7.31 | 6.04 | 6.45 |
| CD129 | 4.42 | 3.26 | 6.46 | 7.64 | 5.74 | 3.05 |
| CD158 | 4.23 | 3.14 | 5.06 | 6.32 | 5.31 | 3.32 |
| CD181 | 4.62 | 3.29 | 5.83 | 6.95 | 5.54 | 3.38 |
| CD193 | 4.36 | 3.21 | 5.79 | 6.98 | 5.55 | 3.65 |
| CD196 | 4.33 | 3.27 | 26.4 | 7.51 | 5.42 | 4.13 |
| CD1d | 4.49 | 3.35 | 6.6 | 8.1 | 5.61 | 3.49 |
| CD20 | 3.5 | 2.56 | 4.81 | 5.89 | 5.21 | 2.86 |
| CD22 | 4.46 | 2.96 | 5.16 | 6.2 | 5.51 | 2.99 |
| CD220 | 4.34 | 3.22 | 7.72 | 8.97 | 5.57 | 3.55 |
| CD235ab | 5.31 | 4.03 | 6.73 | 7.84 | 4.77 | 2.67 |
| CD258 | 4.08 | 3.17 | 6.03 | 7.12 | 5.36 | 3.1 |
| CD274 (PD-L1) | 50.5 | 35.6 | 14.6 | 33.4 | 60.4 | 30.8 |
| CD319 | 4.1 | 3.43 | 5.57 | 7.11 | 5.5 | 3.86 |
| CD32 | 3.46 | 2.7 | 4.91 | 5.79 | 4.85 | 2.97 |
| CD326 (Ep-CAM) | 1462 | 1236 | 474 | 31.6 | 4455 | 2247 |
| CD338 | 15 | 7.77 | 5.86 | 6.3 | 10.5 | 4.11 |
| CD368 | 4.27 | 3.44 | 5.89 | 6.55 | 5.51 | 3.51 |
| CD45RA | 3.82 | 2.98 | 4.89 | 6.18 | 4.87 | 2.71 |
| CD45RB | 3.43 | 2.79 | 5.17 | 5.84 | 4.85 | 2.75 |
| CD49e | 72.2 | 52.9 | 302 | 315 | 42.1 | 49.3 |
| CD52 | 3.58 | 2.91 | 18.8 | 6.09 | 6.04 | 3.04 |
| CD66a/c/e | 4 | 3.58 | 5.1 | 6.28 | 6.13 | 5.87 |
| CD85h | 5.01 | 3.78 | 5.86 | 7.32 | 5.85 | 3.99 |
| CD85j | 3.76 | 3.58 | 5.23 | 6.82 | 5.64 | 2.98 |
| CD86 | 3.83 | 3.11 | 5.02 | 6.05 | 7.43 | 4.72 |
| CD92 | 31.3 | 28.5 | 194 | 173 | 79.7 | 44.5 |
| CXCR7 | 6.48 | 5.1 | 6.33 | 8.35 | 8.03 | 4.44 |
| Delta Opiod Receptor | 3.83 | 3 | 5.67 | 6.86 | 5.31 | 2.83 |
| DRD1 | 3.78 | 3.13 | 5.25 | 5.69 | 5.06 | 3.06 |
| EphA2 | 213 | 49.2 | 326 | 222 | 1015 | 450 |
| FceRIa | 4.14 | 3.06 | 4.96 | 6.23 | 5.02 | 3.15 |
| GARP | 3.69 | 2.93 | 5.1 | 6.17 | 5.06 | 2.95 |
| IL-15Ra | 6.78 | 7.85 | 7.09 | 7.13 | 6.32 | 3.38 |
| LT-βR | 70.1 | 49.9 | 39.1 | 31.9 | 134 | 69.1 |
| MRGX2 | 4.19 | 3.3 | 5.9 | 6.69 | 5.42 | 2.95 |
| TMEM8A | 6.54 | 6.74 | 11 | 19.9 | 15.8 | 10.5 |
| CD254 | 4.28 | 3.81 | 18.1 | 8.51 | 6.85 | 3.74 |
| CD318 | 116 | 87.8 | 193 | 89.1 | 219 | 83 |
| Mouse IgG3, k Isotype Ctrl | 11 | 6.94 | 33.5 | 12.1 | 5.44 | 2.81 |
| CD255 | 15.7 | 8.39 | 12 | 30.5 | 7.92 | 3.89 |
| SSEA-4 | 3.76 | 2.97 | 4370 | 1100 | 53 | 206 |
| Mouse IgM Isotype Ctrl | 3.7 | 3.05 | 6.34 | 6.48 | 4.8 | 2.7 |
| Sialyl Lewis X | 20.7 | 15.6 | 4.87 | 6.41 | 6.56 | 8.62 |
| TRA-1-81 | 3.62 | 2.96 | 15.8 | 7.31 | 5.47 | 2.85 |
| CD160 | 3.72 | 3.03 | 5.01 | 6.11 | 5.19 | 2.71 |
| CD57 | 3.45 | 2.94 | 11.9 | 6.21 | 6.62 | 3.31 |
| CD66b | 3.35 | 2.68 | 4.58 | 5.72 | 4.8 | 2.62 |
| TRA-1-60-R | 3.31 | 2.85 | 20.2 | 6.61 | 4.83 | 2.57 |
| Rat IgG1, k Isotype Ctrl | 4.09 | 3.57 | 6.29 | 7.02 | 5.17 | 2.68 |
| CD115 | 4.87 | 3.9 | 8.67 | 7.5 | 7.05 | 3.51 |
| CD201 | 66.4 | 17.2 | 26.4 | 62.7 | 56.3 | 38.6 |
| Rat IgG2a, k Isotype Ctrl | 3.81 | 3.06 | 5.88 | 6.67 | 4.94 | 2.73 |
| CD120b | 3.99 | 3.27 | 5.77 | 6.42 | 5.23 | 2.86 |
| CD210 | 5.07 | 4.29 | 6.65 | 8.26 | 5.9 | 3.54 |
| CD267 | 3.54 | 2.97 | 5.51 | 6.31 | 4.97 | 2.99 |
| CD294 | 4.5 | 3.46 | 5.78 | 7.08 | 5.81 | 3.37 |
| CD49f | 744 | 386 | 850 | 451 | 565 | 144 |
| CD85a | 3.58 | 2.78 | 8.38 | 5.97 | 5.27 | 2.78 |
| CD85d | 4 | 3.26 | 5.98 | 7.02 | 5.27 | 3.14 |
| IgG Fc | 3.6 | 2.86 | 4.61 | 5.96 | 4.88 | 2.7 |
| Integrin B7 | 3.78 | 2.86 | 4.64 | 6.06 | 5.02 | 2.83 |
| XCR1 | 4.94 | 4.52 | 6.34 | 10 | 4.97 | 2.8 |
| Podoplanin | 94 | 65.8 | 5.06 | 7.7 | 38.8 | 32.5 |
| Rat IgG2b, k Isotype Ctrl | 3.33 | 2.78 | 4.58 | 5.72 | 4.89 | 2.55 |
| CD132 | 4.28 | 3.69 | 7.15 | 7.45 | 5.34 | 3.22 |
| CD195 | 3.73 | 2.97 | 5.52 | 6.35 | 5.97 | 3.01 |
| CX3CR1 | 3.54 | 2.66 | 5.14 | 6.17 | 5.12 | 3.05 |
| Rat IgM, k Isotype Ctrl | 4.86 | 3.78 | 4.85 | 5.78 | 5.15 | 2.85 |
| SSEA-3 | 3.4 | 2.81 | 115 | 13.9 | 5.02 | 5.19 |

Proteins with greater than 2-fold expression of associated isotype controls with either an approximately 2-fold increase in expression (green) or 2-fold decrease in expression (red) in resistant cells when compared to sensitive cells are highlighted.
