# Supplementary material for: Antibody dependent cell-mediated cytotoxicity selection pressure induces diverse mechanisms of resistance
Source: Cancer Biol Ther. 2023 Oct 25;24(1):2269637. doi: 10.1080/15384047.2023.2269637 (PMC10601508; doi:10.1080/15384047.2023.2269637)
Supplement: Supplemental Material [file KCBT_A_2269637_SM9645.docx]

**Supplementary Table 1: Reverse Phase Protein Array Analytes**

| **Protein/Phospho-protein Identity** |
| --- |
| 4EBP1 S65 |
| 4EBP1 T70 |
| Acetyl CoA Carboxylase S79 |
| AKT S473 (D9E) |
| AKT T308 |
| ALK Y1586 |
| ALK Y1604 |
| AMPKa1 S485 |
| Androgen Rec S650 |
| Androgen Rec S81 |
| AR-V7 total |
| ASK1 S83 |
| ATM S1981 |
| ATP Citrate Lyase S454 |
| ATR S428 |
| Aurora A T288/B T232/C T198 |
| BAD S112 |
| BAD S136 |
| BCL-2 S70 |
| B-RAF S445 |
| BRCA1/2 S1524 |
| cABL T735 |
| Caspase 3, cleaved D175 |
| Caspase 9, cleaved D330 |
| Catenin, beta S33/S37/T41 |
| Catenin, beta T41/S45 |
| CD3 epsilon total |
| Chk-1 S345 |
| cKIT Y703 |
| Cofilin S3 |
| cPLA2 S505 |
| C-RAF S338 |
| CREB S133 |
| Cyclin A1 total |
| Cyclin B1 total |
| Cyclin D1 total |
| EGFR total |
| EGFR Y1068 |
| EGFR Y1148 |
| EGFR Y1173 |
| EGFR Y992 |
| eIF4G S1108 |
| ELK1 S383 |
| eNOS S113 |
| eNOS S1177 |
| eNOS/NOS III S116 |
| ERK1/2 T202/Y204 |
| Estrogen Rec alpha S118 |
| Estrogen Rec alpha total |
| FADD S194 |
| FAK Y576/577 |
| FGF Rec Y653/Y654 |
| FOXM1 T600 |
| FOXO1 S256 |
| FOXO1 T24/FOXO3a T32 |
| FOXO3a S253 |
| GSK3aB S21/S9 |
| H2A.X S139 |
| HER2 total |
| HER2 Y1248 |
| HER2 Y877 |
| HER3 total |
| HER3 Y1289 |
| HER4 total |
| HER4 Y1284 |
| Heregulin total |
| Histone H3 S10 |
| HLA-DR total |
| HLA-DR/DP/DQ/DX total |
| HSP90a T5/T7 |
| IGF1R Y1131/Insulin Rec Y1146 |
| IGF1R Y1135/Y1136-Insulin Res Y1150/Y1151 |
| IkBa S32/S36 |
| Insulin Rec beta total |
| IRS1 S612 |
| IRS1 total |
| JAK1 Y1022/Y1023 |
| Ki67 total |
| LC3B total |
| LKB1 S334 |
| M-CSF Rec Y723 |
| MDM2 S166 |
| MEK1/2 S217/S221 |
| MET Y1234/1235 |
| MLH1 total |
| MSH2 total |
| MSK1 S360 |
| mTOR S2448 |
| NFkB p65 S536 |
| NRF2 total |
| p27 T187 |
| p38 MAPK T180/Y182 |
| p53 S15 |
| p53 total |
| p70S6K S371 |
| p70S6K T389 |
| p70S6K T412 |
| p90RSK S380 |
| p90RSK T359/S363 |
| PAK1 S199/S204-PAK2 S192/S197 |
| PAK1 T423/PAK2 T402 |
| PARP, cleaved D214 |
| PDGFRb Y716 |
| PDGFRb Y751 |
| PDK1 S241 |
| PD-L1 total (22C3) |
| PI3K p85 Y458/p55 Y199 |
| PKA C T197 |
| PKC alpha S657 |
| PLCgamma1 Y783 |
| PP2A alpha subunit total |
| PRAS40 T246 |
| PRK1 T774/PRK2 T816 |
| PTEN S380 |
| PTEN total |
| Raf S259 |
| Ras-GRF1 S916 |
| Rb S780 |
| RET Y905 |
| Ron Y1353 |
| RSK3 T356/S360 |
| S6RP S235/S236 |
| S6RP S240/S244 |
| SAPK/JNK T183/Y185 |
| SGK1 S78 |
| SHC Y317 |
| SMAD2 S245/S250/S255 |
| Src Family Y416 |
| SRC Y527 |
| STAT1 Y701 |
| STAT2 Y690 |
| STAT3 S727 |
| STAT3 Y705 |
| STAT4 Y693 |
| STAT5 Y694 |
| STAT6 Y641 |
| TROP2 total |
| Tuberin/TSC2 Y1571 |
| TYK2 Y1054/Y1055 |
| VEGFR2 Y1175 |
| VEGFR2 Y996 |
| YAP S127 |
