# Supplementary material for: Antibody dependent cell-mediated cytotoxicity selection pressure induces diverse mechanisms of resistance
Source: Cancer Biol Ther. 2023 Oct 25;24(1):2269637. doi: 10.1080/15384047.2023.2269637 (PMC10601508; doi:10.1080/15384047.2023.2269637)
Supplement: Supplemental Material [file KCBT_A_2269637_SM9588.docx]

**
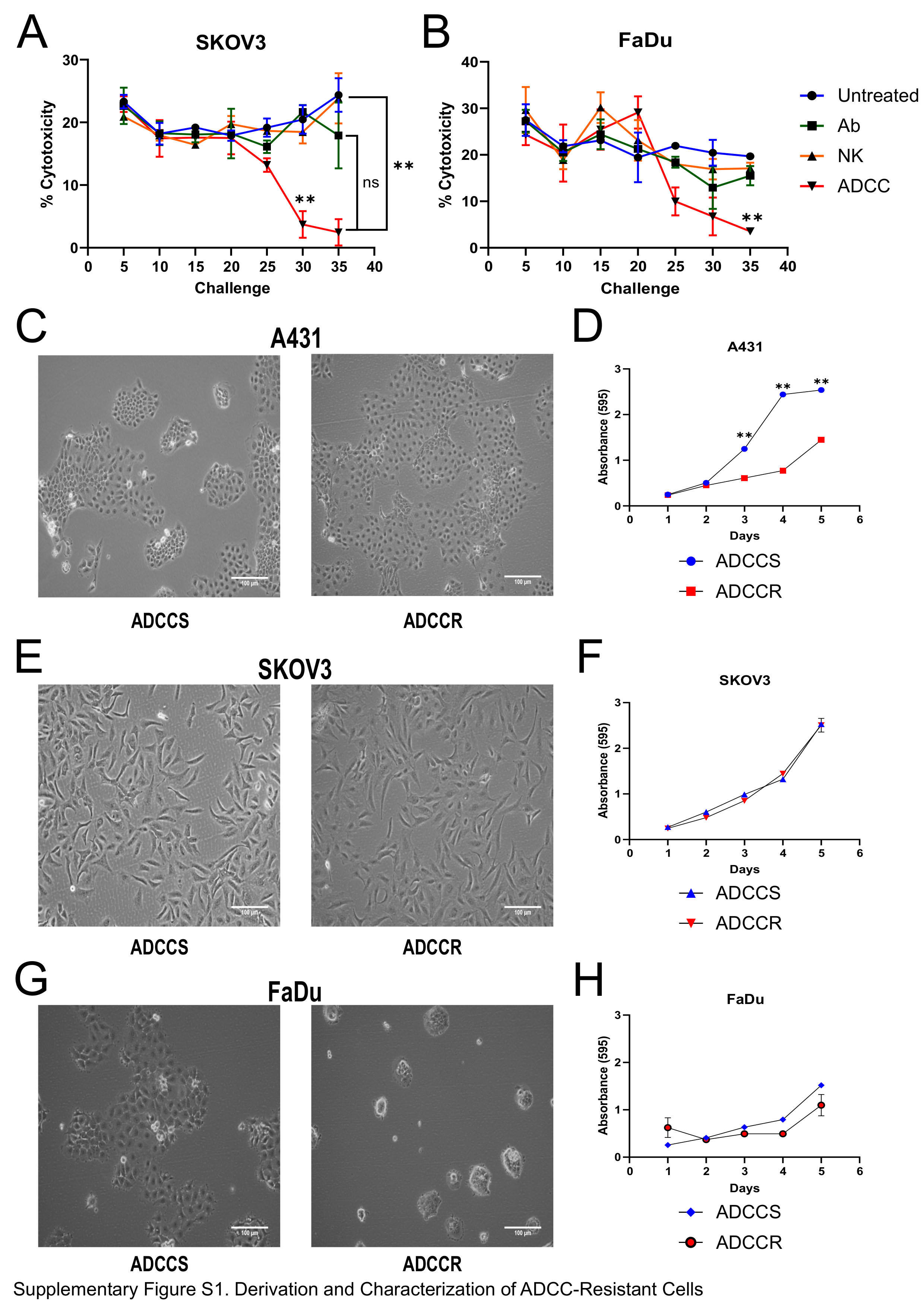
**

**Supplementary Figure S1. Derivation and Characterization of ADCC-Resistant Cells**

**A,** Percent cytotoxicity of untreated (Blue), antibody treated (Green), NK92-CD16V treated (Orange), and ADCC condition treated (Red) SKOV3 cells at every 5 challenges during derivation of resistance as measured by ADCC Assay (n=2 for Challenges 5-25, n=3 for Challenges 30 and 35). Unpaired two-tailed *t-*test, ns, not significant, **, *P*<0.01. Error bars, SEM. **B,** Percent cytotoxicity of untreated (Blue), antibody treated (Green), NK92-CD16V treated (Orange), and ADCC condition treated (Red) FaDu cells at every 5 challenges during derivation of resistance as measured by ADCC Assay (n=2 for Challenges 5-30, n=3 for Challenge 35). Unpaired two-tailed *t-*test, **, *P*<0.01. Error bars, SEM. **C,** A431 cells were seeded in T-175 flasks and imaged after 96hrs for morphological characterization. Representative light microscopy images of A431 ADCC sensitive (ADCCS) and ADCC resistant (ADCCR) cells. Representative of n=2, magnification 20x, scale bar = 100µm. **D,** *In vitro* proliferation of ADCC-resistant (ADCCR) compared to ADCC-sensitive (ADCCS) A431 cells over 5 days in absence of ADCC conditions as measured by crystal violet staining. Unpaired two-tailed *t-*test, **, *P*<0.01. Error bars, SEM. **E,** SKOV3 cells were seeded in T-175 flasks and imaged after 96hrs for morphological characterization. Representative light microscopy images of SKOV3 ADCC-sensitive (ADCCS) and ADCC-resistant (ADCCR) cells. Representative of n=2, magnification 20x, scale bar = 100µm. **F,** *In vitro* proliferation of ADCC-resistant (ADCCR) compared to ADCC-sensitive (ADCCS) SKOV3 cells over 5 days in absence of ADCC conditions as measured by crystal violet staining. Error bars, SEM. **G,** FaDu cells were seeded in T-175 flasks and imaged after 96hrs for morphological characterization. Representative light microscopy images of FaDu ADCC-sensitive (ADCCS) and ADCC-resistant (ADCCR) cells. Representative of n=2, magnification 20x, scale bar = 100µm. **H,** *In vitro* proliferation of ADCC-resistant (ADCCR) compared to ADCC-sensitive (ADCCS) FaDu cells over 5 days in absence of ADCC conditions as measured by crystal violet staining. Error bars, SEM.

**
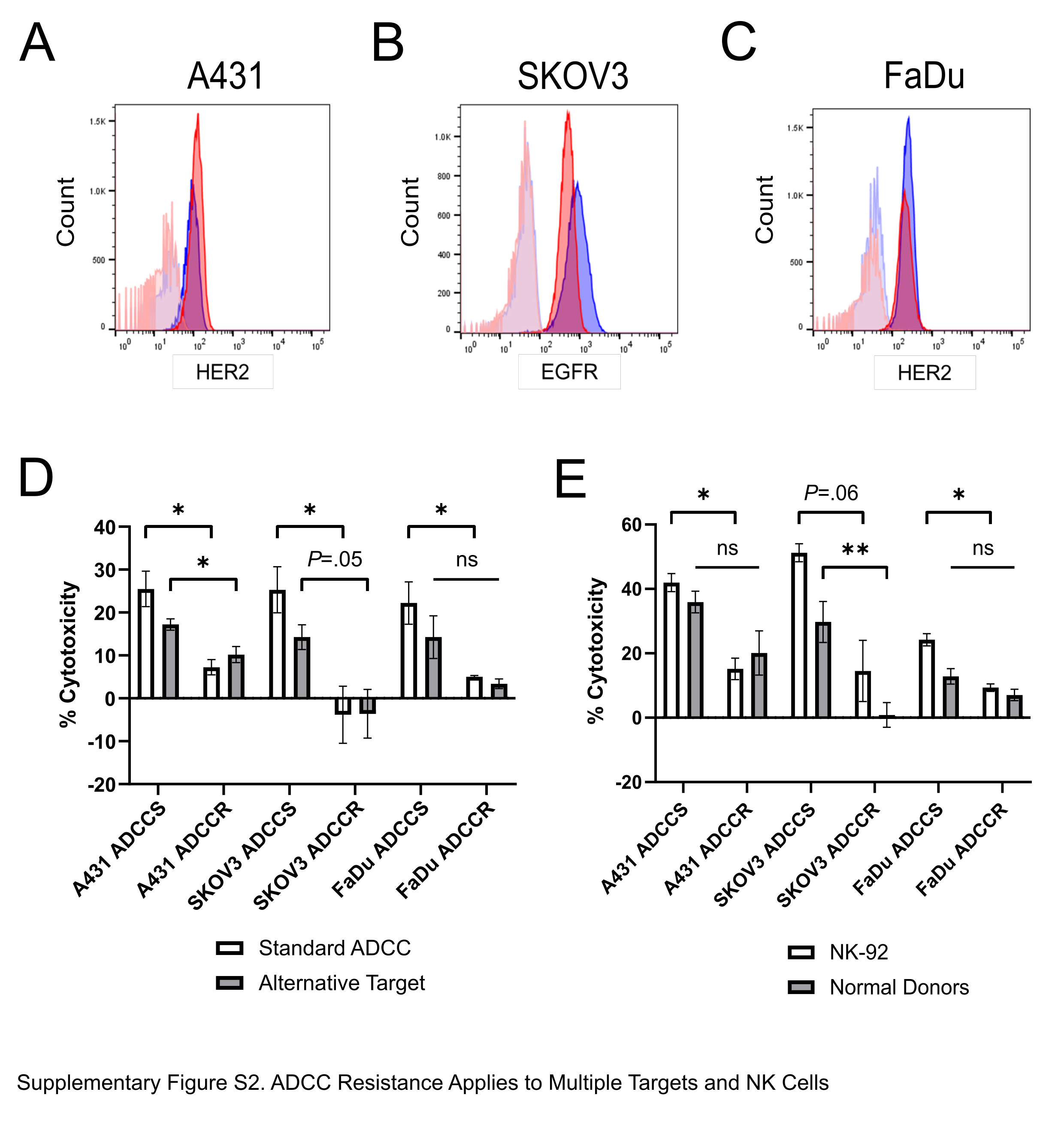
**

**Supplementary Figure S2: ADCC Resistance Applies to Multiple Targets and NK Cells**

**A-C,** Surface HER2 or EGFR expression measured by flow cytometry in ADCC-sensitive (Blue) and ADCC-resistant (Red) A431 (A), SKOV3 (B), and FaDu (C) cells with isotype control staining in ADCC-sensitive (light blue) and ADCC-resistant (light red) cells. Representative histograms from n=2 experiments each. **D,** Percent cytotoxicity of each ADCC-sensitive and ADCC-resistant cell line as measured by ADCC assay when using standard ADCC conditions (white bar) or when using an alternative target (HER2/Trastuzumab in A431/FaDu and EGFR/Cetuximab in SKOV3) (gray bar) (n=2). Unpaired two-tailed *t*-test, ns, not significant. Error bars, SEM. **E,** Percent cytotoxicity of each ADCC-sensitive and ADCC-resistant cell line as measured by ADCC assay when using NK-92 (white bar) (n=2), or healthy donor NK cells (gray bar) (n=10; two experimental replicates of five different healthy donors) as effector cells. Unpaired two-tailed *t*-test, *, *P*<.05, Error bars, SEM.

**
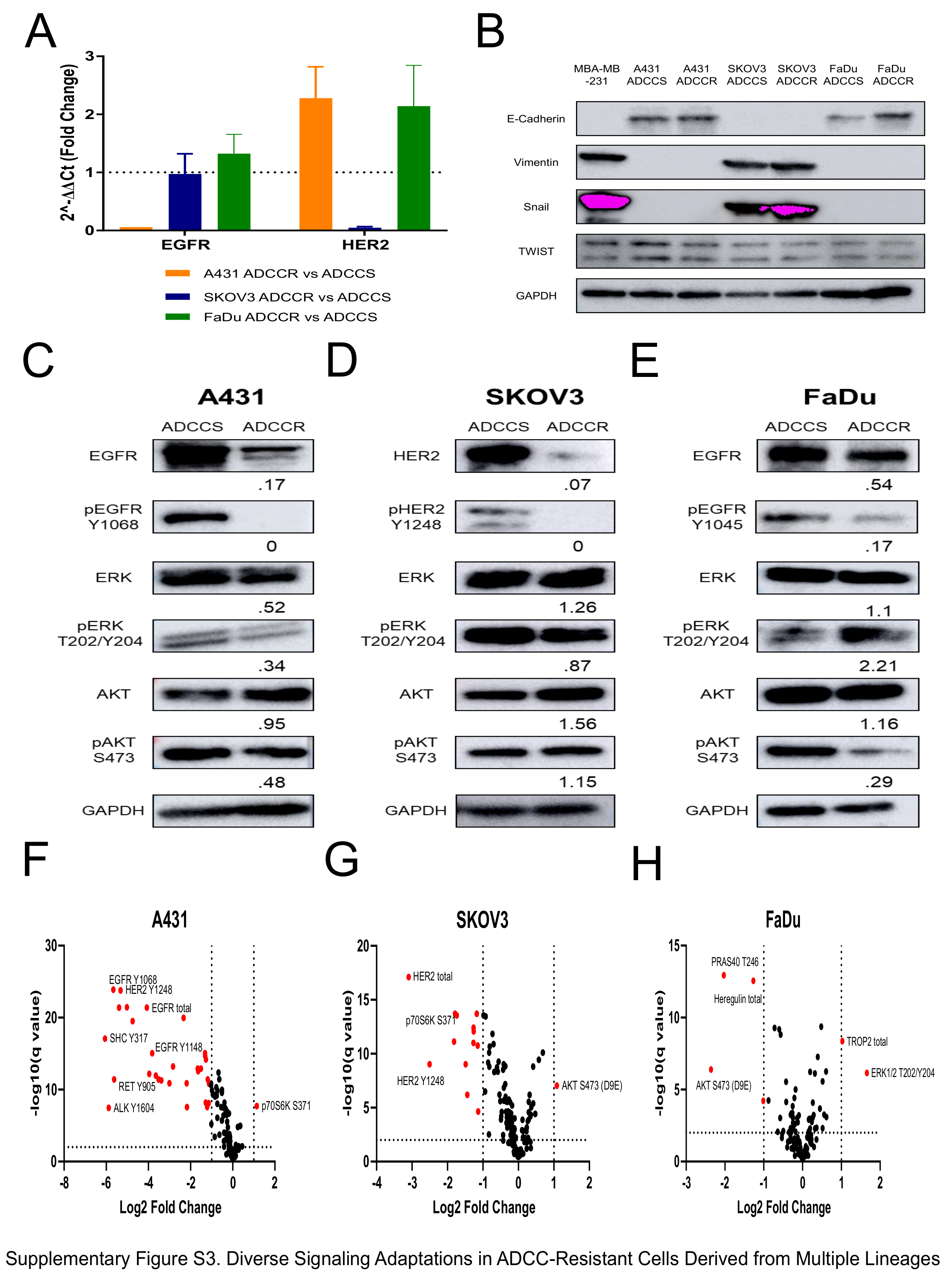
**

**Supplementary Figure S3: Diverse Signaling Adaptations in ADCC-Resistant Cells Derived from Multiple Lineages**

**A,** Fold change in EGFR and HER2 gene expression in ADCC-resistant compared to ADCC-sensitive A431 (Orange), SKOV3 (Blue), and FaDu (Green) cells as determined by RT-qPCR (n = 2). **B,** Western blot analysis of EMT markers in ADCC-sensitive (ADCCS) and ADCC-resistant (ADCCR) A431, SKOV3, and FaDu cells. MBA-MB-231 cells were used as positive control for EMT marker expression. **C-E,** Western blot analysis of EGFR or HER2 signaling pathways in ADCC-sensitive (ADCCS) and ADCC-resistant (ADCCR) A431 (C), SKOV3 (D), and FaDu (E) cells. Densitometry values for expression normalized for GAPDH relative to expression in ADCCS cells indicated below for each protein. **F-H,** Volcano plot of differential protein and phospho-protein expression in ADCC-resistant compared to ADCC-sensitive A431 (F), SKOV3 (G), and FaDu (H) cells. Multiple unpaired *t*-tests were performed on 12 replicates of each cell line. Proteins and phospho-proteins with significantly upregulated or downregulated expression and beyond the –log10(q) value threshold of ±2 and the log2 fold change threshold of ±1 are highlighted in red.

**
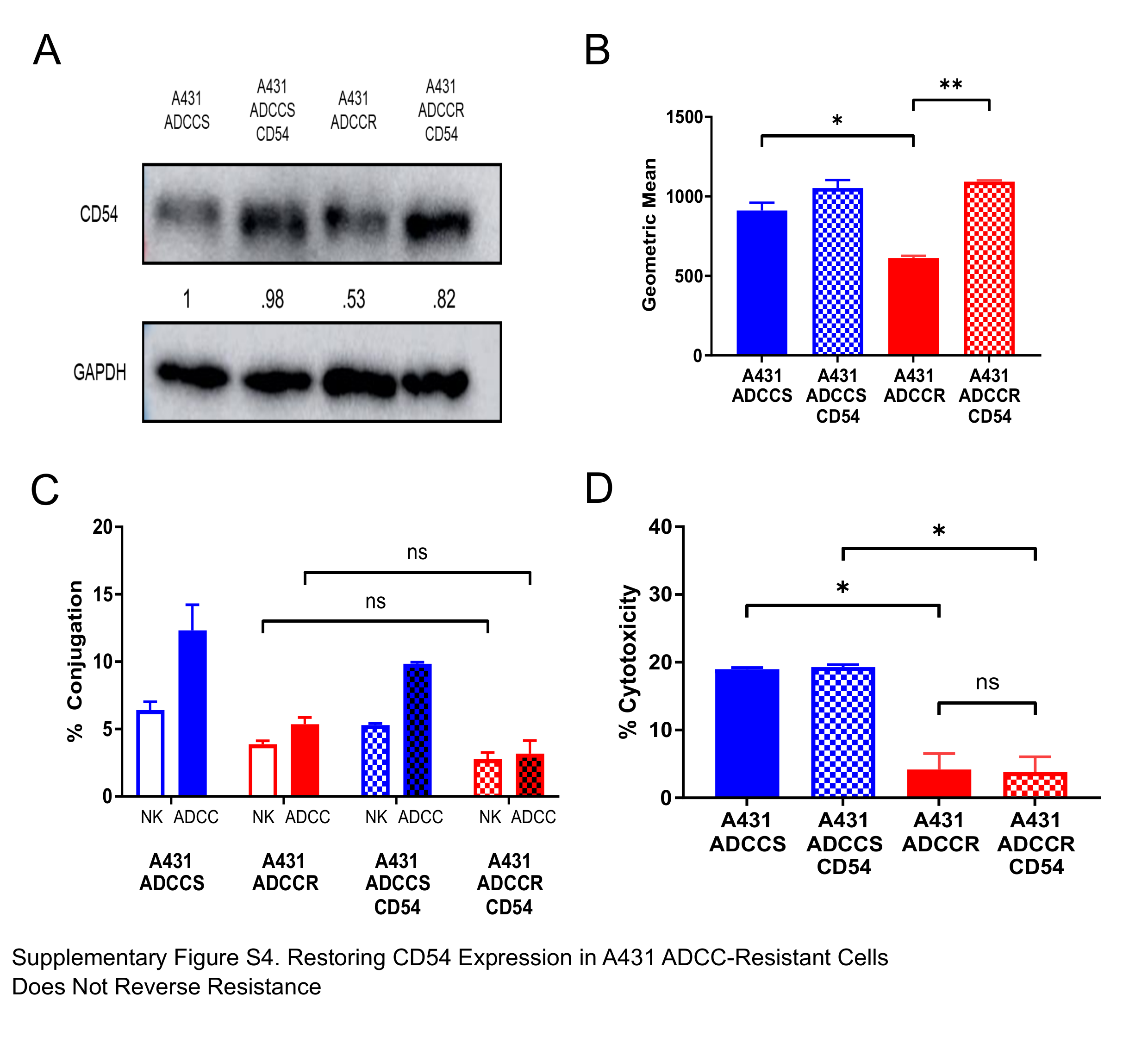
**

**Supplementary Figure S4: Restoring CD54 Expression in A431 ADCC Resistant Cells Does Not Reverse Resistance**

**A,** Western blot analysis of CD54 protein expression in ADCC-sensitive (ADCCS) and ADCC-resistant (ADCCR) A431 cells with and without transduction of CD54 expression plasmid. Densitometry values for expression normalized for GAPDH relative to A431 ADCCS indicated below. **B,** Geometric mean of CD54 expression as measured by flow cytometry in ADCC-sensitive (blue) compared to ADCC-resistant (red) A431 cells with (checkered bars) and without (solid bars) transduction of CD54 expression plasmid (n=2). Unpaired two-tailed *t*-test, *, *P*<.05, **, *P*<.01. Error bars, SEM. **C,** Percent of NK92-CD16V cell-target cell conjugates in ADCC-sensitive (ADCCS, Blue) compared to ADCC-resistant (ADCCR, Red) A431 cells when treated with NK92-CD16V cells alone (open bar) or ADCC conditions (full bar) after 2hrs with (checkered bars) and without (solid bars) transduction of CD54 expression plasmid (n=2). Unpaired two-tailed *t-*test, ns, not significant. Error bars, SEM. **D,** Percent cytotoxicity of ADCC-sensitive (ADCCS, Blue) compared to ADCC-resistant (ADCCR, Red) A431 cells with (checkered bars) and without (solid bars) transduction of CD54 expression plasmid as measured by ADCC Assay (n=2). Unpaired two-tailed *t*-test, ns, not significant, *, *P*<0.05. Error bars, SEM.

**
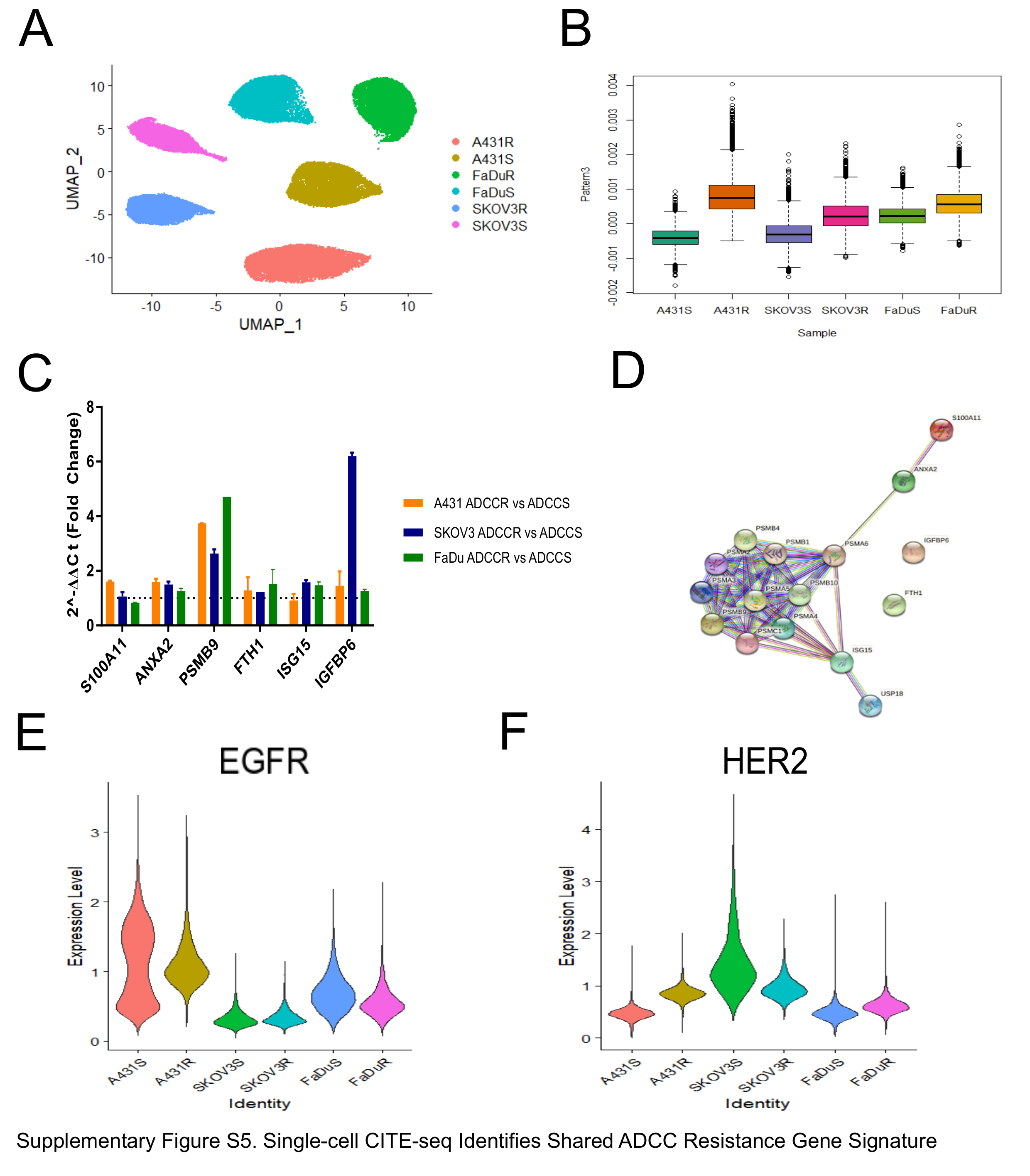
**

**Supplementary Figure S5: Single-cell CITE-seq Identifies Shared ADCC Resistance Gene Signature**

**A,** Overall UMAP plot demonstrating each ADCC-sensitive and -resistant cell line presents a specific gene expression profile. **B,** Boxplots indicating association between each cell line with the previously identified ADCC resistance gene signature composed of IFN-response and DNA-damage response genes (Pattern3) by COGAPS analysis. **C,** Fold change in gene expression for the six upregulated genes in ADCC-resistant compared to ADCC-sensitive A431 (Orange), SKOV3 (Blue), and FaDu (Green) cells as determined by RT-qPCR (n = 2). **D,** STRING protein-protein interacting network analysis of the six upregulated genes shared in all ADCC resistant cells. **E and F,** Violin plot of EGFR (E) and HER2 (F) protein expression level in each ADCC-sensitive and -resistant cell line measured by scCITE-seq.

**
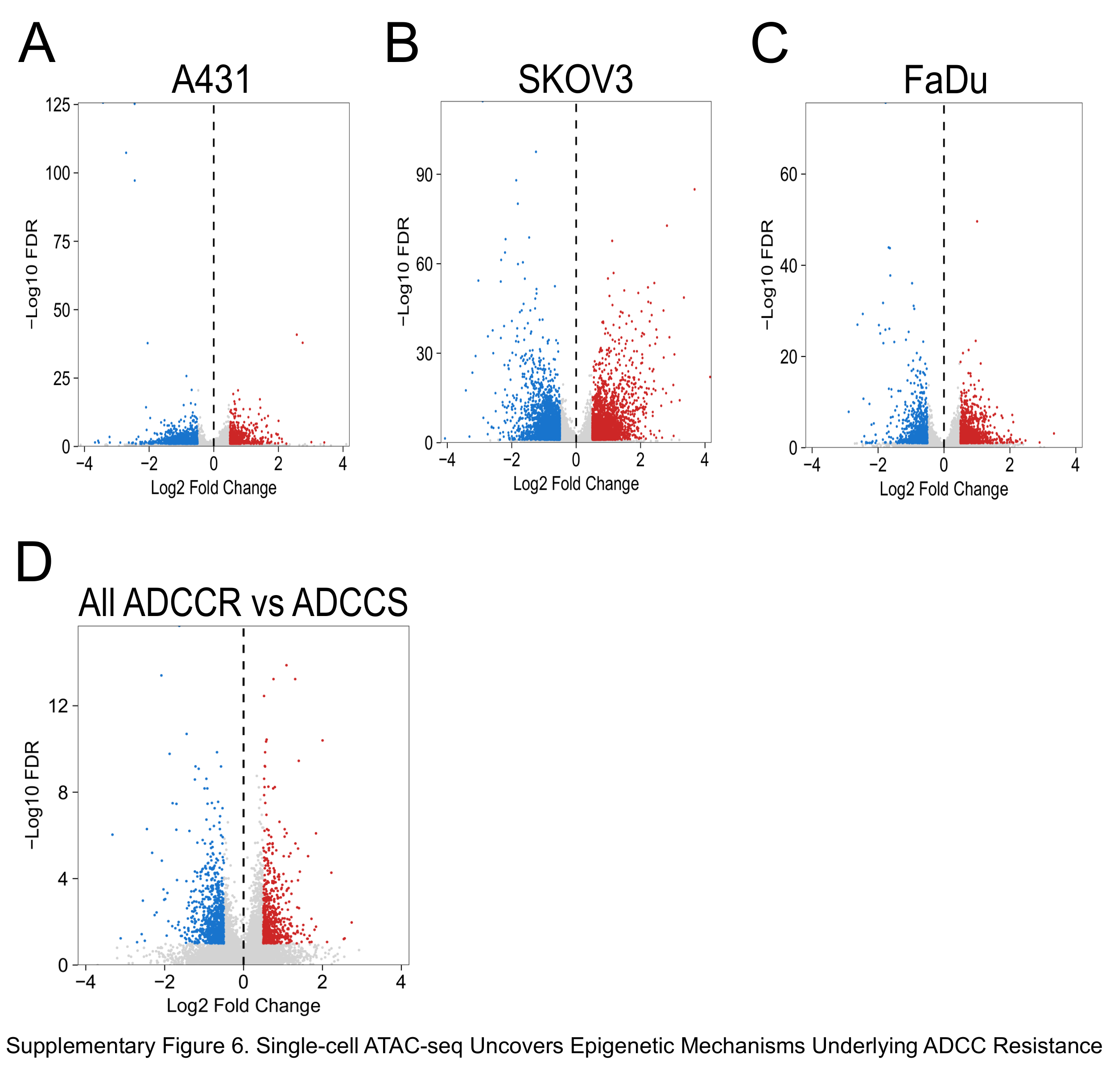
**

**Supplementary Figure S6: Single-cell ATAC-seq Uncovers Epigenetics Mechanisms Underlying ADCC Resistance**

**A-C,** Volcano plots of genes with differential chromatin accessibility scores between A431 (A), SKOV3 (B), and FaDu (C) ADCC-sensitive and ADCC-resistant cells. Genes with significantly upregulated accessibility are marked in red whereas genes with significantly downregulated accessibility are marked in blue. Genes with –Log10(FDR)>2 and log2 fold change ±0.5 were considered significant. **D,** Volcano plot of genes with shared differential chromatin accessibility scores across all ADCC-sensitive (ADCCS) and ADCC-resistant (ADCCR) cell lines. Genes with significantly upregulated accessibility are marked in red whereas genes with significantly downregulated accessibility are marked in blue. Genes with –Log10(FDR)>2 and log2 fold change ±0.5 were considered significant.

**
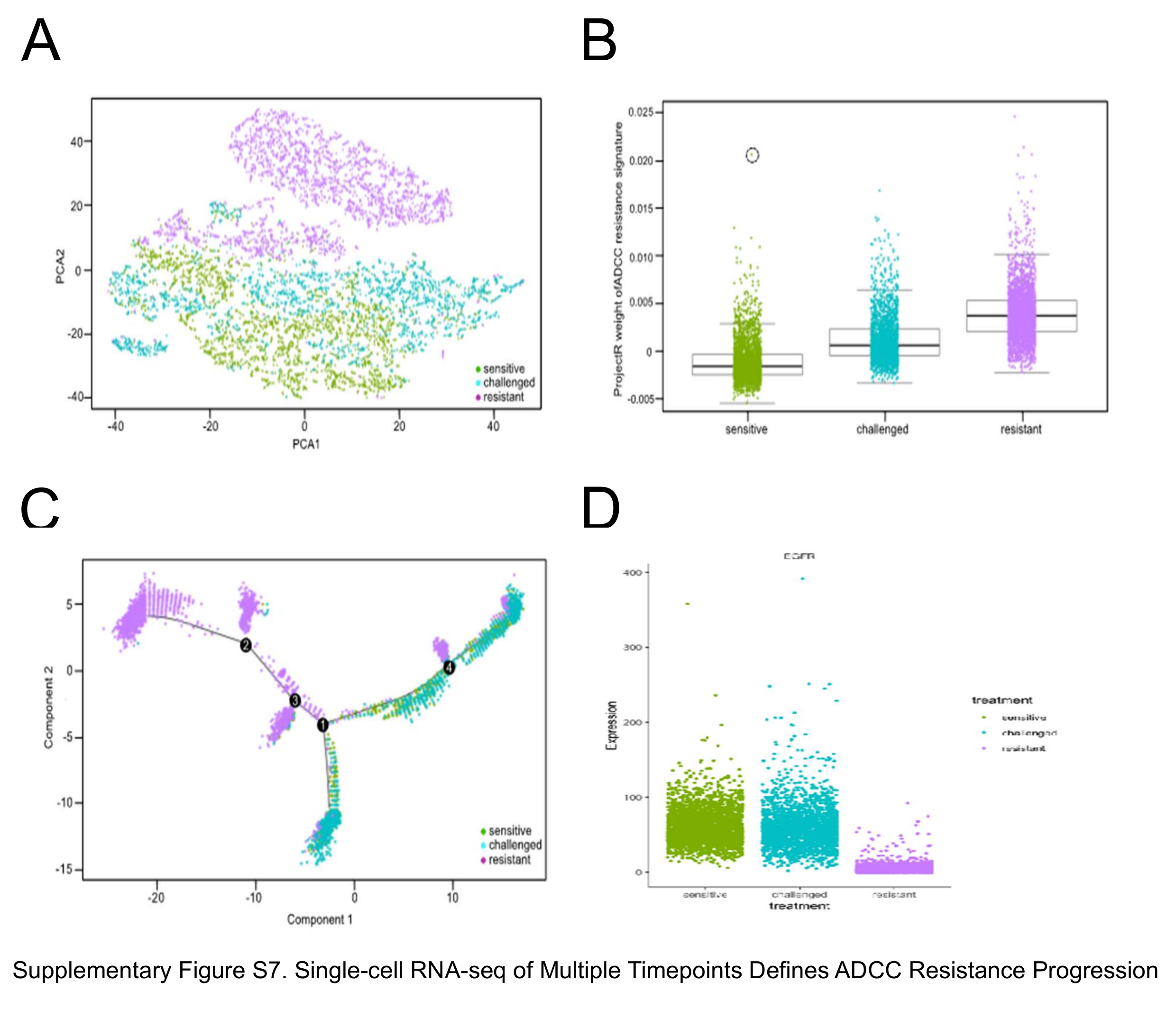
**

**Supplementary Figure S7: Single-cell RNA-seq of Multiple Timepoints Defines ADCC Resistance Progression**

**A,** Clustering of the A431 ADCC-sensitive, ADCC-challenged, and ADCC-resistant cell lines by PCA. **B,** ProjectR transfer of previously published bulk RNA resistance signature, showing single subclone within the sensitive cells with resistance signature (circled). **C,** Pseudotime analysis defining progression of ADCC resistance by PCA. **D,** Gene expression of *EGFR* in individual A431 ADCC-sensitive, ADCC-challenged, and ADCC-resistant cell lines.

**
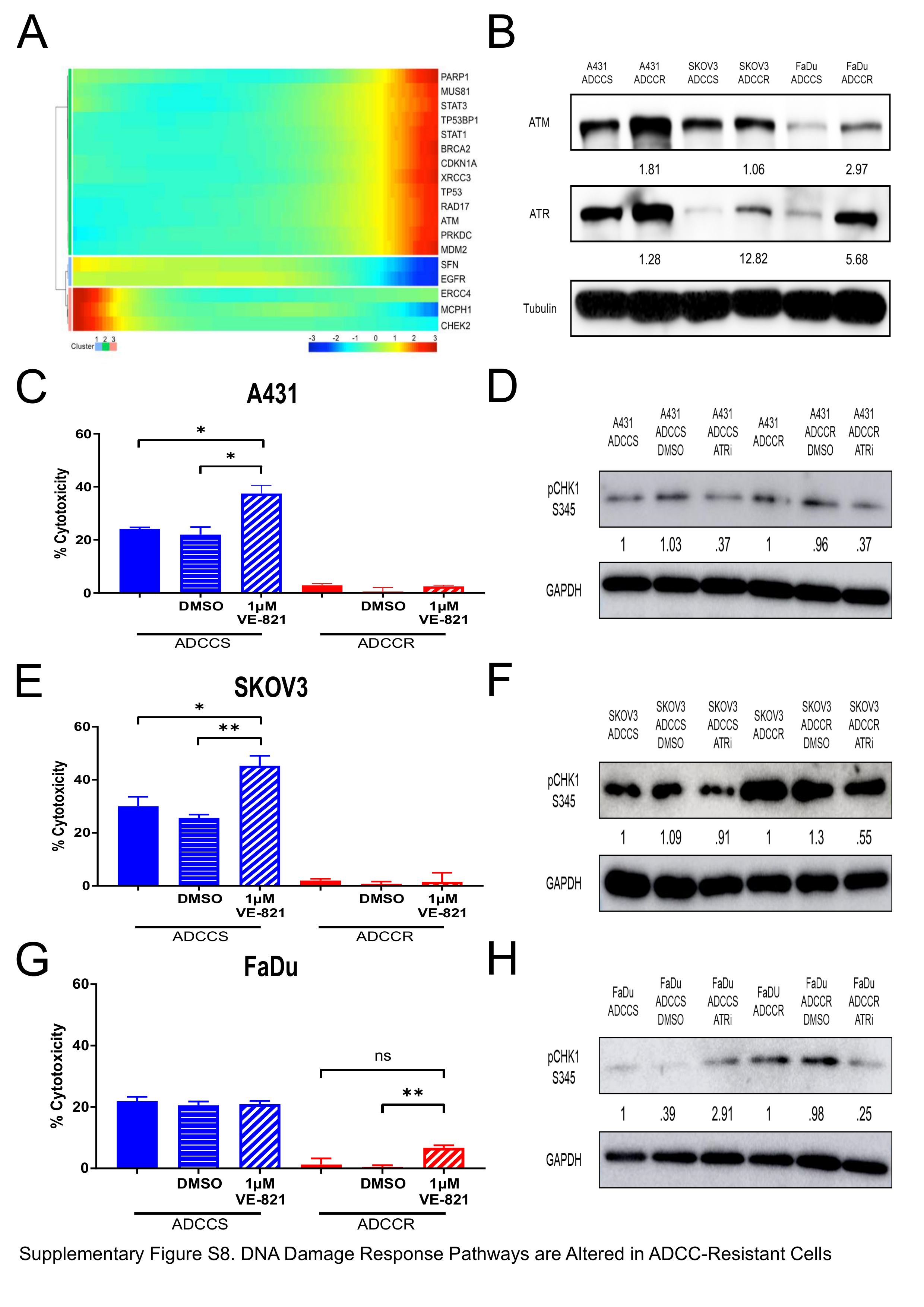
**

**Supplementary Figure S8: DNA Damage Response Pathways are Altered in ADCC-Resistant Cells**

**A,** Heatmap of DNA damage response network gene expression measured by scRNA-seq of parental ADCC-sensitive A431 cells, A431 cells after one ADCC challenge, and fully ADCC-resistant A431 cells as a function of pseudotime. **B,** Western blot analysis of ATM and ATR protein expression in ADCC-sensitive (ADCCS) and ADCC-resistant (ADCCR) A431, SKOV3, and FaDu cells. Densitometry values for expression normalized to GAPDH in ADCC-resistant relative to ADCC-sensitive cells are indicated. **C,** Percent cytotoxicity of ADCC-sensitive (Blue) and ADCC-resistant (Red) A431 cells as measured by ADCC assay when cells are pretreated with either medium alone (solid bars) or medium supplemented with DMSO (horizontal hash bars) or 1μM of the ATR kinase inhibitor VE-821 (diagonal hash bars) for 1hr. Wells are washed prior to ADCC assay (n=3). Unpaired two-tailed *t*-test, *, *P*<.05. Error bars, SEM. **D,** Representative western blot analysis of ATR kinase target CHK1 S345 in A431 cells treated with either medium alone or medium supplemented with DMSO or 1μM of the ATR kinase inhibitor VE-821 for 1hr. Densitometry values for expression normalized to GAPDH in cells treated with DMSO or VE-821 compared to untreated cells are indicated. **E,** Percent cytotoxicity of ADCC-sensitive (Blue) and ADCC-resistant (Red) SKOV3 cells as measured by ADCC assay when cells are pretreated with either medium alone (solid bars) or medium supplemented with DMSO (horizontal hash bars) or 1μM of the ATR kinase inhibitor VE-821 (diagonal hash bars) for 1hr. Wells are washed prior to ADCC assay (n=3). Unpaired two-tailed *t*-test, **, *P*<.01, *, *P*<.05. Error bars, SEM. **F,** Representative western blot analysis of ATR kinase target CHK1 S345 in SKOV3 cells treated with either medium alone or medium supplemented with DMSO or 1μM of the ATR kinase inhibitor VE-821 for 1hr. Densitometry values for expression normalized to GAPDH in cells treated with DMSO or VE-821 compared to untreated cells are indicated. **G,** Percent cytotoxicity of ADCC-sensitive (Blue) and ADCC-resistant (Red) FaDu cells as measured by ADCC assay when cells are pretreated with either medium alone (solid bars) or medium supplemented with DMSO (horizontal hash bars) or 1μM of the ATR kinase inhibitor VE-821 (diagonal hash bars) for 1hr. Wells are washed prior to ADCC assay (n=3). Unpaired two-tailed *t*-test, ns, not significant, **, *P*<.01. Error bars, SEM. **H,** Representative western blot analysis of ATR kinase target CHK1 S345 in FaDu cells treated with either medium alone or medium supplemented with DMSO or 1μM of the ATR kinase inhibitor VE-821 for 1hr. Densitometry values for expression normalized to GAPDH in cells treated with DMSO or VE-821 compared to untreated cells are indicated.
